# Small Molecules from Nature Targeting G-Protein Coupled Cannabinoid Receptors: Potential Leads for Drug Discovery and Development

**DOI:** 10.1155/2015/238482

**Published:** 2015-11-17

**Authors:** Charu Sharma, Bassem Sadek, Sameer N. Goyal, Satyesh Sinha, Mohammad Amjad Kamal, Shreesh Ojha

**Affiliations:** ^1^Department of Internal Medicine, College of Medicine and Health Sciences, United Arab Emirates University, P.O. Box 17666, Al Ain, Abu Dhabi, UAE; ^2^Department of Pharmacology and Therapeutics, College of Medicine and Health Sciences, United Arab Emirates University, P.O. Box 17666, Al Ain, Abu Dhabi, UAE; ^3^Department of Pharmacology, R. C. Patel Institute of Pharmaceutical Education & Research, Shirpur, Mahrastra 425405, India; ^4^Department of Internal Medicine, College of Medicine, Charles R. Drew University of Medicine and Science, Los Angeles, CA 90059, USA; ^5^King Fahd Medical Research Center, King Abdulaziz University, Jeddah, Saudi Arabia; ^6^Enzymoics, 7 Peterlee Place, Hebersham, NSW 2770, Australia

## Abstract

The cannabinoid molecules are derived from *Cannabis sativa* plant which acts on the cannabinoid receptors types 1 and 2 (CB_1_ and CB_2_) which have been explored as potential therapeutic targets for drug discovery and development. Currently, there are numerous cannabinoid based synthetic drugs used in clinical practice like the popular ones such as nabilone, dronabinol, and Δ^9^-tetrahydrocannabinol mediates its action through CB_1_/CB_2_ receptors. However, these synthetic based *Cannabis* derived compounds are known to exert adverse psychiatric effect and have also been exploited for drug abuse. This encourages us to find out an alternative and safe drug with the least psychiatric adverse effects. In recent years, many phytocannabinoids have been isolated from plants other than *Cannabis*. Several studies have shown that these phytocannabinoids show affinity, potency, selectivity, and efficacy towards cannabinoid receptors and inhibit endocannabinoid metabolizing enzymes, thus reducing hyperactivity of endocannabinoid systems. Also, these naturally derived molecules possess the least adverse effects opposed to the synthetically derived cannabinoids. Therefore, the plant based cannabinoid molecules proved to be promising and emerging therapeutic alternative. The present review provides an overview of therapeutic potential of ligands and plants modulating cannabinoid receptors that may be of interest to pharmaceutical industry in search of new and safer drug discovery and development for future therapeutics.

## 1. Introduction

The endocannabinoid system (ECS), an important lipid signaling and immunomodulator system, has begun to reap attention as it is widely involved in modulating host of physiological responses ranging from appetite, respiration, metabolism, inflammation, pain, neurotransmission, and so forth. The ECS is comprised of the G-protein coupled receptors (GPCRs) such as cannabinoid receptors 1 and 2 (CB_1_ and CB_2_); cannabinoid receptor ligands also known as endocannabinoids are characterized by arachidonyl ethanolamide (anandamide, AEA) and 2-arachidonoyl glycerol (2-AG) [1, 2] and the enzymes involved in synthesis and degradation of the endocannabinoids. The levels of the endocannabinoids in the tissues are maintained by the critical balance between their biosynthesis (involving phospholipase D and diacylglycerol lipase-dependent and other pathways) and cellular uptake as well as degradation by the enzymes: fatty acid amide hydrolase (FAAH) and/or monoacylglycerol lipases (MAGL) [3]. Recently, some additional GPCRs such as GPR18, GPR55, and GPR119 have been recognized as members of the cannabinoid family; however the physiological significance is yet to be established [4].

The CB_1_ and CB_2_ receptors are well characterized members of the GPCR which couple to G-proteins in the G_i/o_ family. The activation of the CB_1_ and CB_2_ receptors causes the numerous intracellular effects which may be cell type and ligand specific and involve the inhibition of various voltage gated Ca^+2^ channels and adenylate cyclase activity and the activation of K^+^ channels, resulting in lower levels of cAMP along with activation of MAPK pathways [5]. The CB_1_ receptors regulate the activities of adenylyl cyclase, ERK, glycogen synthase kinase 3, and calcium and potassium channels [5]. The CB_2_ receptor couples to G_i_ to mediate their cellular effects via inhibition of adenylyl cyclase and regulation of transcription factors [5]. The inhibition of activation of cannabinoid receptors and inhibition of endocannabinoid degradative enzymes have been found to enhance endocannabinoid signaling and harness the therapeutic potential of the ECS as an important therapeutic target [6, 7].

In recent years, research is focusing on the unique neuromodulator system, ECS, which is named after the plant that led to its discovery [3]. The pervasive and varied regulatory actions of the ECS in maintenance of general health and diseases have supported the regulatory approval of several molecules of natural and synthetic origin as novel drugs that modulate the cannabinoid receptor signaling mediated by CB_1_ or CB_2_ receptors or alter the ECS activity by reducing the endocannabinoid tone by inhibiting FAAH and MAGL [6, 8]. The potential role for ECS-based therapies must be explored with a clear and complete picture of the potential beneficial and adverse effects that will occur from exogenous activation and/or inhibition of ECS using cannabinoid based medicines. The modulation of ECS by cannabinoid based medicines holds remarkable therapeutic promise in a variety of pathological conditions including neuropathic pain, diabetic complications, obesity, stroke, hypertension, cancer, psychosis, glaucoma, epilepsy, addiction, and neurodegenerative diseases including Alzheimer's disease, multiple sclerosis, and Parkinson's disease [7, 9].

The cannabinoids comprise compounds that produced endogenous (endocannabinoids), synthetic, and active components of* Cannabis sativa*, a traditional source of about 100 natural cannabinoids also known as phytocannabinoids [10]. The physiological effects of these phytocannabinoids derived from* Cannabis sativa* have been known since ancient times and used for both leisure and medicinal purposes and have generated immense interest for pharmaceutical development. Phytocannabinoids are defined as agents of plant origin that interacts with either of cannabinoid receptors or shares chemical similarity with cannabinoids or both. It is known that they arise from the interaction of Δ^9^-tetrahydrocannabinol (Δ^9^-THC), the main psychoactive constituent of the plant;* Cannabis sativa* interact with cannabinoid receptors [11–13].

Several classes of synthetic cannabinoid agents have been developed for the therapeutic targeting of the several components of ECS. Among them, rimonabant (SR141716A; Acomplia), a CB_1_ receptor antagonist/inverse agonist, makes a therapeutic success for the management of obesity but was withdrawn because of safety concerns about its psychiatric adverse effects, particularly increased incidence of depression, anxiety, and suicidal tendencies [10]. Numerous illicitly produced synthetic cannabinoid agonists typically acting as agonists at CB_1_ receptors that mimic the effects of Δ^9^-THC have been reported to drug monitoring agencies. Synthetic agents produce atypical pharmacological effects such as hypertension, seizures, and panic attacks. This is explained by atypical effect of CB_1_ receptor agonist, which is apparently higher for synthetic cannabinoids: JWH-018 and JWH-073 compared with Δ^9^-THC, the agent mainly accountable for the behavioral effects of cannabis [14].

In parallel to the development of synthetic analogues modulating ECS components, the pharmaceutical companies followed several approaches to target the cannabinoid receptors and modulate ECS activity including the development of phytocannabinoid compounds isolated from the plants. Currently, several drugs which modulate the CB_1_ or CB_2_ receptors are at present in the clinic such as Cesamet (nabilone), Marinol (dronabinol; Δ^9^-THC), and Sativex (cannabidiol and Δ^9^-THC). The agents, nabilone and dronabinol, are indicated to relieve chemotherapy-induced nausea and vomiting. Dronabinol is also used as appetizer, while the plant derived cannabis preparation. Sativex is frequently indicated for the symptomatic relief of neuropathic pain in adults with multiple sclerosis and spasticity and is also used as an adjunct to relieve pain in adult patients with advanced cancer.

The potential agents derived from plants targeting ECS have become a central focus of contemporary translational research for diverse indications with important unmet medical demands. The present review focuses on medicinal plants that have shown to modulate the ECS appearing as therapeutic possibility for diseases which involves ECS dysregulation. The present review focuses on natural small molecules, isolated and characterized as cannabinoid receptors modulator. These naturally derived molecules could offer the potential leads for future drug discovery and the targeting of endocannabinoid dysregulation or the diseases where endocannabinoid modulation represents an important therapeutic target. Additionally, the medicinal plants modulating ECS are also provided that can be subjected for the isolation of components possessing cannabinoid receptor agonist or antagonist activity. The actions of cannabinoid compounds partly involve several non-CB receptor dependent mechanisms and are regarded as an additive beneficial effect of phytocannabinoids molecules for multitargeting.

## 2. Phytochemicals as Lead Compounds Targeting ECS

**Figure 1:**
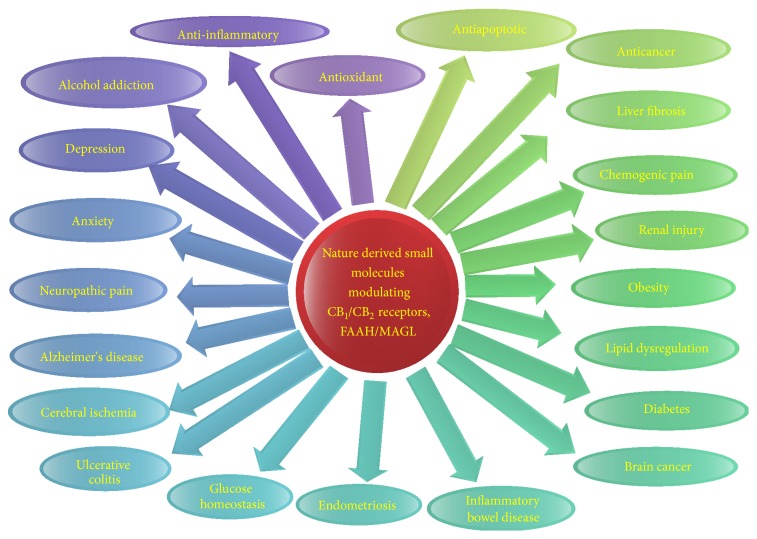
Cannabinoid receptor mediated medicinal and pharmacological activities of lead compounds isolated from medicinal plants.

Following the progress in chemical isolation and screening techniques, several novel lead molecules were isolated and characterized from the natural products for the development of new drugs. In current years, numerous molecules have been isolated and characterized which showed cannabinoid receptor affinity, efficacy, and therapeutic benefits in the* in vitro*,* in silico*, and* in vivo* studies [15–21]. The agents were also found to inhibit endocannabinoid metabolizing enzymes, FAAH, DAGL, and MAGL inhibitors, and exhibit their potential efficacy mediated by the cannabinoid mediated mechanism [7].  Figure 1 depicts the cannabinoid receptors and endocannabinoid metabolizing enzymes mediated pharmacological effects and therapeutic benefits of small molecules derived from nature.

Directly acting ligands are the compounds which exhibit high binding affinities (in low nanomolar to micromolar range) to the cannabinoid receptors and exert distinct functional effects behaving either as agonists, inverse agonists, partial agonists, or antagonist [22], whereas indirectly acting ligands target either the key proteins in the ECS which regulate endocannabinoid levels in tissues or the allosteric sites on the CB_1_ receptors [6]. Recently, availability of different tools such as radioligand and [^35^S]GTP*γ*S binding assays facilitated the characterization of agonists, antagonists, and inverse agonists for cannabinoid receptors. Some practical guidelines and specific considerations in order to characterize the ligands using these assays are available for cannabinoid receptors. The agonists which bind to CB_1_ and CB_2_ receptors show little selectivity; however the CB_1_ and CB_2_ receptor antagonists are highly selective usually in nanomolar affinity at the respective receptor. This allows differentiating the CB_1_ or CB_2_ mediated mechanism and responses of* in vitro* and* in vivo* studies. In addition to the selective CB_1_ and CB_2_ antagonists that are used to block agonist effects, there are also genetic tools (CB_1_/CB_2_ receptor knockout mice) available to the research community. There are several nonselective agonists which are available which prefer either CB_1_ or CB_2_ receptors [4, 10].

In this review, the small molecules derived from natural products targeting ECS components are described in order to provide them as standard sources of templates for developing novel ligands for pharmaceutical development and clinical usage. The database searches using Medline/PubMed, EMBASE, Google Scholar, and Science Direct were conducted to include all the available published literature in the present review paper. The years of coverage for literature retrieval were from 1975 to May 25, 2015. The search was limited to English language publications; however if the abstract was available in English, then it is included in the present paper. For literature search, the standard MeSH such as natural products, cannabinoid receptor modulators, cannabinoid agents, medicinal plants, and cannabinoid ligands and articles all together on cannabinoid ligands were used in the database search engines. In almost all cases, the original articles were obtained and the relevant data was extracted.

**Figure 2:**
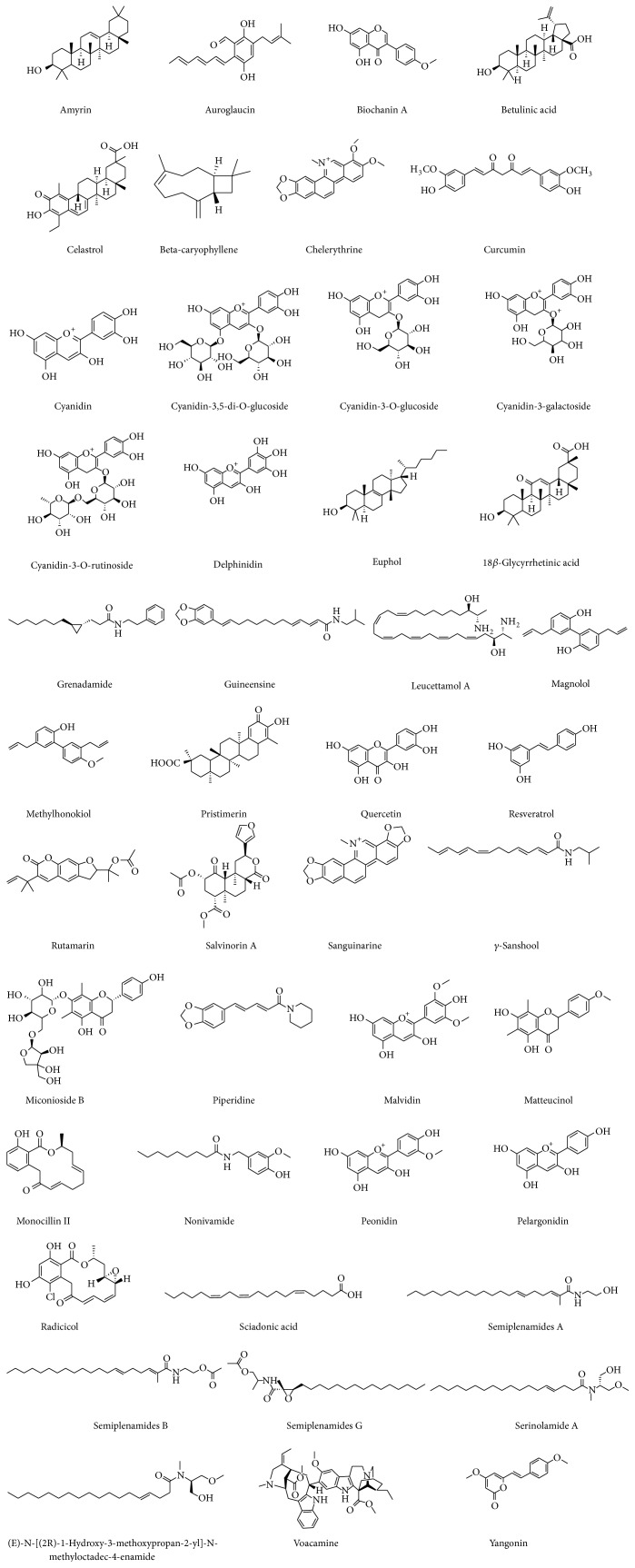
Chemical structure of isolated phytochemicals targeting endocannabinoid system.

Table 1 depicts the physicochemical properties and drug likeness of phytochemicals and Figure 2 represents the chemical structure of phytochemicals modulating cannabinoid receptors and endocannabinoid metabolizing enzymes.  Table 2 shows the therapeutic properties and underlying cannabinoid mediated mechanism of small natural molecules modulating cannabinoid receptors and endocannabinoid metabolizing enzymes. The cannabinoids are chemically defined as terpenoalcoholic compounds and chemical class of molecules identified till date is provided in Table 3. Recently, some selective full agonists and antagonists for specific CB_1_ and CB_2_ receptors have been recognized. Among the phytocannabinoids, *β*-caryophyllene is one which has been identified as a full agonist for CB_2_ receptors and isolated from cannabis as well as noncannabis plant [18]. This generated interest in characterizing the cannabinoid-like compounds or CB receptor modulating ligands from plants other than cannabis, which is considered a traditional source of phytocannabinoids.

### 2.1. Alkylamides Derivatives

#### 2.1.1. Alkylamides from* Echinacea angustifolia*

Various studies have demonstrated that the CB_2_ receptors are primarily found in immune cells and participate in immune regulation [16, 17, 23, 24]. Thus, interactions of alkylamides with CB_2_ receptors can be demonstrated by immunomodulatory effect of* Echinacea* preparations [21, 25, 26]. Two alkylamides, dodeca-2E,4E,8Z,10Z-tetraenoic acid isobutylamide and dodeca-2E,4E-dienoic acid isobutylamide, have been isolated from* Echinacea purpurea* and* Echinacea angustifolia* [21, 27]. Chemically, alkylamides show structural similarity with anandamide and bind with CB_2_ receptors more potently than endogenous cannabinoids with the *K*_*i*_ values (CB_2_ approximately 60 nM; CB_1_ > 1500 nM) and act as full agonist on CB_2_ receptors in nanomolar range. Also, the molecular modeling studies have shown that alkylamide compounds bind in the solvent-accessible cavity in CB_2_ receptors which is directed by the H-bonding and pi-pi interactions [27]. Furthermore, these compounds raised total intracellular Ca^2+^ in CB_2_-positive promyelocytic HL 60 cells as demonstrated by abrogation of the effects by SR144528 and also inhibit the enzyme, FAAH [27]. Though, the ketolactones found in* Echinacea pallida* (purple cornflower) did not show cannabinoid activity [28]. Another alkylamide, undeca-2-ene-8,10-diynoic acid isolated from* Echinacea* spp., stimulates 3T3-L1 differentiation mediated by PPAR-*γ* activity demonstrating that anti-inflammatory property of alkylamides is due to polyvalent activity [29, 30].

#### 2.1.2. Alkylamides from* Otanthus maritimus* L

Several alkylamides have been isolated from dichloromethane root extract of* Otanthus maritimus* L. (family: Asteraceae), an aromatic herb growing on sandy beaches along the Mediterranean coasts. These compounds exhibit cannabinoid receptors binding affinity as demonstrated in the* in vitro*,* in silico*, and* in vivo* studies [15, 31]. The* in silico* studies were carried out by generating 3D models of hCB_2_ receptors in homology modeling [31]. The root extract showed high binding affinity to CB_1_ and CB_2_ receptors with *K*_*i*_ values of 2.2 *μ*g/mL and 1.3 *μ*g/mL, respectively, and moderate affinity to *μ*- and *δ*-opioid receptors in radioligand assay. Among the several identified compounds from extract, a tertiary alkylamide, 1-[(2*E*,4*E*,8*Z*)-tetradecatrienoyl] piperidine, showed most potent binding affinity with both CB_1_ and CB_2_ receptors with a *K*_*i*_ value of 0.8 *μ*M and 0.16 *μ*M, respectively. It showed CB_2_ selectivity with a *K*_*i*_CB_1_/*K*_*i*_CB_2_ = 5, with significant potency (*K*_*i*_ = 160 nM) [31]. Other isolated alkylamides as dodeca-2*E*,4*E*-dienoic acid isobutylamide, tetradeca-2*E*,4*E*-dienoic acid isobutylamide, tetradeca-2*E*,4*E*,8*Z*-trienoic acid isobutylamide, and 1-[(2*E*,4*E*,8*Z*)-tetradecatrienoyl] piperidine showed highest affinity for CB_2_ receptors and show less affinity to opioid receptors. In regard to CB_2_ receptor affinity, the structure activity relationship (SAR) studies reveal the influence of double bonds geometry in dodecatetraenoic acid isobutylamides. The alkylamides, N-substituted with an isobutyl or dimethylbutyl group and represented by a secondary alkylamide as the amide part, appear to be involved in the CB_2_ receptor interaction [32]. However, it is observed that the tertiary amide 1-[(2*E*,4*E*,8*Z*)-tetradecatrienoyl] piperidine which contains a piperidinyl moiety linked to a C14 acyl chain appears to have more affinity and potency on CB_2_ than dodeca-2*E*,4*E*-dienoic acid isobutylamide, an active principle of* Echinacea* species [15]. Overall, alkylamides from* Echinacea* and* Otanthus* spp. appear to be a good source of CB_2_ receptors ligands in drug discovery.

### 2.2. *α*,*β*-Amyrin

The pentacyclic triterpene and mixture (1 : 1) of two isomers, *α*,*β*-amyrin, are mainly constituent of the resin of* Protium kleinii* and* Protium heptaphyllum*. The CB receptor mediated anti-inflammatory and antinociceptive effect of *α*,*β*-amyrin has been shown in mice model of neuropathic pain [33]. It reduced mechanical and thermal hyperalgesia and inflammation induced by complete Freund's adjuvant and by partial sciatic nerve ligation in animal models. The antinociceptive responses were mediated by activation of the ECS and comparable to the synthetic molecules, ACEA and JWH-133. The reversals of antinociceptive effects by CB_1_ or CB_2_ receptor antagonists (AM251 and AM630, resp.) as well as knockdown of the CB_1_/CB_2_ gene demonstrate CB activity. It binds to CB_1_ receptors with a high affinity (*K*_*i*_ = 0.133 nM) and to CB_2_ receptors with a lower affinity (*K*_*i*_ = 1989 nM) along with absence of behavioral disturbances. The binding to CB_1_ receptors was 200–300-fold more potent than Δ^9^-THC. However, in contrast to Δ^9^-THC and 2-AG, *α*,*β*-amyrin showed an unusual 15000-fold more binding selectivity for CB_1_ receptors over CB_2_. Furthermore, *α*,*β*-amyrin decreased proinflammatory cytokines and chemokines and prevented activation of the transcriptional factors: NF-*κ*B and cyclic adenosine monophosphate response element binding (CREB) and the expression of cyclooxygenase-2 (COX-2) in footpads and spinal cords of mice. It also prevented upregulation of CB_2_R mRNA but failed to affect CB_1_ receptor mRNA upregulation as well as cortical levels of both CB_1_ and CB_2_ receptors. In another study, Chicca et al. [34] showed CB receptor binding interactions of *α*,*β*-amyrin using hCB_1_/hCB_2_ receptors transfected CHO-K1 cells and its effects on the endocannabinoid transport in U937 cells. The study showed that it did not bind to cannabinoid receptors (*K*_*i*_ > 10 *μ*M) whereas it inhibited 2-AG hydrolysis in pig brain homogenates and failed to inhibit AEA. Additionally, *β*-amyrin is found to weakly inhibit human MAGL in a rapid, reversible, and noncompetitive manner, similar to structurally related but more potent triterpene, pristimerin. Subsequently, Matos et al. [35] also showed the cannabimimetic activity of *α*,*β*-amyrin in dextran sulfate sodium-induced colitis in mice by diminishing disease activity, colonic damage, and activity of myeloperoxidase, N-acetylglucosaminidase, and attenuating induction of proinflammatory mediators: cytokines, chemokines, and adhesion molecules in the colon. The abrogation of the beneficial effects of *α*,*β*-amyrin by CB_1_ receptor blocker, but not by CB_2_ receptor blocker, demonstrates the CB_1_ receptor mediated mechanism. Additionally, *α*,*β*-amyrin treatment reduced the MAGL and FAAH enzymes. Integrating the ECS modulatory properties *α*,*β*-amyrin seem to be a promising candidate for future therapeutics.

### 2.3. Anthocyanins

Anthocyanins are water-soluble polyphenol compounds abundantly found in colored fruits and vegetables particularly in red and blue fruits such as blueberry, cranberry, and red cabbage. These have been shown to regulate several intracellular functions. Numerous studies have shown that anthocyanins and anthocyanidins exhibit antioxidant, redox-inflammatory signaling which contributes to its analgesic, cardioprotective, neuroprotective, anticancer, atherogenic, antihyperlipidemic, and antihypertensive effects. The cannabinoid receptor activity has been demonstrated by competitive radioligand assays of cyanidin (*K*_*i*_ = 16.2 *μ*M) and delphinidin (*K*_*i*_ = 21.3 *μ*M) for hCB_1_ receptors whereas similar affinities for CB_2_ receptors have been shown by cyanidin (*K*_*i*_ = 33.5 *μ*M), delphinidin (*K*_*i*_ = 34.3 *μ*M), and peonidin (*K*_*i*_ = 46.4 *μ*M) [36]. However, the cyanidin derivatives such as cyanidin-3,5-di-O-glucoside, cyanidin-3-O-glucoside, cyanidin-3-O-galactoside, cyanidin-3-O-rutinoside, malvidin, and pelargonidin showed inhibition of both CB_1_ and CB_2_ receptors. Additionally, cyanidin-3-O-*β*-glucoside also reported to activate all forms of PPARs and reduces hepatic lipids by altering the expression of genes involved in lipid metabolic pathways. Taking altogether the multiple pharmacological properties, anthocyanins appear as polypharmacological agent for diseases involving dysregulation of ECS and PPARs [36].

### 2.4. Auroglaucin

Auroglaucin, a benzaldehyde compound, is obtained from ethyl acetate extract of fungus* Eurotium repens* collected from Tifton, GA. The extract as well as auroglaucin showed binding affinity for CB_1_ (62.6%) and CB_2_ receptors (43.1%) using CP55,940 assay in CHO-K1 cells [37]. The extract also showed affinity with opioid receptors with binding affinity more than 40%. The IC_50_ for CB_1_ and CB_2_ receptor was 15.2 and 19.9 *μ*M, respectively [37].

### 2.5. Betulinic Acid

Betulinic acid is a widely distributed pentacyclic triterpenoid with a lupan skeleton in the plant kingdom. Betulinic acid isolated from the extract of several plants and its synthetic analogues exhibit a broad spectrum of activities including antioxidant, anti-inflammatory, antiangiogenic, immunomodulatory, and anticancer. Liu et al. [38] investigated the effects of CB_1_ and CB_2_ receptor antagonists AM251 and AM630, respectively, on betulinic acid-dependent repression of Sp1, Sp3, and Sp4 and survivin. Betulinic acid and either AM251 or AM630 attenuated the effects of betulinic acid persuaded downregulation of Sp1, Sp3, and Sp4 and survivin and AM251 and AM630 inhibited betulinic acid-mediated downregulation of ErbB2, p-ErbB2, p-MAPK, p-Akt, and YY1 in BT474 and MDA-MB-453 cells. Further, betulinic acid competitively bound to both cannabinoid receptors with *K*_*i*_ values of 36.7 ± 4.1 and 41.2 ± 12.1 *μ*mol/L for mCB_1_ and hCB_2_ receptors, respectively, in radioligand binding assay. The role of CB receptor mediated activity was further confirmed in CB_1_ and CB_2_ knockdown mice partially reversed betulinic acid-induced downregulation of Sp1, Sp3, and Sp4. Betulinic acid-mediated repression of Sp1, Sp3, Sp4, and Sp-regulated genes found because of induction of the Sp repressor ZBTB10 and downregulation of microRNA-27a, which constitutively inhibits ZBTB10 expression, showed that the effects of betulinic acid were CB_1_ and CB_2_ receptor dependent. Further, it has also been shown to activate PPAR-*γ*, which encourages it as a multitargeted agent for future therapeutics.

### 2.6. Biochanin A

Biochanin A is an O-methylated isoflavone compound predominantly found in vegetable plants, red clover, soy, alfalfa sprouts, peanuts, and chickpea, and possesses potent antioxidant, anti-inflammatory, phytoestrogenic, and antineoplastic activities. It showed modest effects on CB_1_ and CB_2_ receptors in [^3^H]CP55,940 assay and inhibited brain CB_1_ receptors (27%) and recombinant CB_2_ receptors (33%) [39]. No studies are available to demonstrate its other activities such as PPAR-*γ* modulation. It has been reported to inhibit FAAH (IC_50_ = 0.62 *μ*M) at micromolar potencies in RBL2H3 cells [39].

### 2.7. *β*-Caryophyllene

*β*-Caryophyllene, a volatile sesquiterpene, is abundantly found in essential oil of many plants such as cloves, oregano, cinnamon, black pepper, hemp, rosemary, and hops [18]. It is popularly used in food, cosmetics, and fragrances as a preservative, additive, and flavoring agent. It is approved by several food and flavor regulatory agencies including United States Food and Drug Administration (FDA) for its use as a food additive and classified as a “generally regarded as safe” compound. Gertsch et al. [18] first time reported that the fractionation of cannabis essential oil yields *β*-caryophyllene which possesses an affinity for CB_2_ receptors. In radioligand assays, (*E*)-*β*-caryophyllene and its isomer (*Z*)-*β*-caryophyllene dose-dependently displaced CP55,940 from hCB_2_ receptors significantly expressed in HEK293 cells (*K*_*i*_ = 155 ± 4 nM) in the nanomolar range and exhibit selective full agonism on CB_2_ receptors. (E)-*β*-caryophyllene exerts potent cannabimimetic anti-inflammatory effects in mice. Several studies have shown the CB_2_ receptor dependent therapeutic effects in ulcerative colitis [40], alcohol addiction [41], cerebral ischemia [42, 43], insulin resistance [44], glutamate neurotoxicity [45], hypertriglyceridemia [46], renal injury [47], liver fibrosis [48], anxiety and depression [49], neuropathic pain [50], Alzheimer's disease [51], and CB_2_ receptor knockout mice [47]. Taking together the cannabimimetic [18], opioidergic [52], and PPARs mediated activity [53], *β*-caryophyllene appears as most promising molecule of pharmaceutical interest with multifunctional and polypharmacological properties.

### 2.8. Catechins

Catechins are the group of polyphenol compounds abundantly found in the leaves of tea, the most popular beverage consumed worldwide and in many fruits and legumes. Catechins are known to maintain heath and general well-being and pharmacotherapeutic effects. The catechin compounds include (−)-epigallocatechin-3-O-gallate (EGCG), (−)-epicatechin-3-O-gallate (ECG), (−)-epigallocatechin (EGC), (−)-epicatechin, and (+)-catechin. These compounds have been comprehensively studied and shown to possess antioxidant, anti-inflammatory, GABAergic, glutamatergic, monoaminergic, opioidergic, and nitrergic modulatory activities and contribute to the several therapeutic benefits. For the first time, Korte et al. [54] evaluated the affinities of EGCG, ECG, EGC, (−)-epicatechin, and (+)-catechin for human CB_1_ and CB_2_ receptors in competitive radioligand binding assays in Chem-1 and CHO cells. All the compounds, namely, EGCG (*K*_*i*_ = 33.6 mM), EGC (*K*_*i*_ = 35.7 mM), and ECG (*K*_*i*_ = 47.3 mM) exhibited binding with CB_1_ and CB_2_ receptors in a dose-dependent manner. However, the weaker binding to CB_2_ receptor was found with inhibition constants more than 50 mM for ECC and EGC. The epimers such as (+)-catechin and (−)-epicatechin in radioligand assays showed slight affinities for both CB_1_ and CB_2_ receptors. The study demonstrates that catechins possess a moderate affinity for CB_1_ receptors whereas binding to CB_2_ receptor was not very prominent. In SAR studies, the ungallated catechins were found to have negligible bioactivities for CB_1_ and CB_2_ and the 3^1^,4^1^,5^1^-trihydroxyl substitution in the catechin B-ring partially contributing to antioxidant, apoptosis-inducer, and *β*-secretase inhibiting activity of catechins did not appear responsible for binding with cannabinoid receptors. Thus, the multifunctional effects of catechins could be further exploited for cannabinoid activities that with additional pharmacological properties may synergize the actions.

### 2.9. Celastrol

Celastrol, a quinone methide triterpenoid, is a pharmacologically active constituent from the root of* Tripterygium wilfordii* and* Celastrus regelii* (family: Celastraceae) also known as Thunder of God Vine in the Asian continent. It is used as a remedy of inflammatory and autoimmune diseases along with its antioxidant, anti-inflammatory, anticancer, and insecticidal activities. Celastrol showed cannabinoid mediated therapeutic activity in inflammatory and neuropathic pain induced by carrageenan and spared nerve injury in animal models [55]. It produces a dose-dependent inhibition of edema and allodynia evidenced by inhibition of inflammatory cytokines and hypersensitivity of nociceptive response. Further, the reversal of antihyperalgesic effects of celastrol by SR144528, a specific CB_2_ receptor antagonist, but not by SR141716, a specific CB_1_ receptor antagonist, demonstrates the analgesia effects of celastrol through CB_2_ signaling. Although celastrol shows an effect on CB_2_ receptors in neuropathic pain and inflammation, further studies would explore its potential as a novel candidate for pain relief.

### 2.10. Chelerythrine and Sanguinarine

Chelerythrine and sanguinarine are the alkaloids of quaternary benzophenanthridine class in several medicinal plants and reported as a potent protein kinase C (PKC) inhibitor. These compounds showed to modify behavior mediated by CB_1_ receptors [56]. The CB_1_ receptor modulatory property of chelerythrine was first reported in a chronic constriction sciatic nerve injury model of neuropathic pain [57]. The application of chelerythrine was found to inhibit CB_1_ receptors mainly within the ipsilateral superficial spinal cord dorsal horn mediating tyrosine kinase receptors. Chelerythrine also inhibits desacetyl levonantradol-dependent activation of CB_1_ receptor in the neuroblastoma cells (N18TG2) and this was supported with modulation of a downstream PKC by CB_1_ receptor [58]. The pseudobase forms of chelerythrine and sanguinarine inhibit CB_1_ receptors similar to Δ^9^-THC at low micromolar concentrations in mouse brain membrane [59]. In [^3^H]CP55,940 binding assay, the IC_50_ of sanguinarine and chelerythrine appears in the 1-2 *μ*M range, which has similar potency like cannabidiol, virodhamine, various Δ^8^-THC derivatives, and certain bicyclic resorcinols [60]. However, these were found weaker than Δ^9^-THC and Δ^9^-tetrahydrocannabivarin, which inhibit the binding of [^3^H]CP55,940 at low nanomolar concentrations [61]. Chelerythrine and sanguinarine showed lesser potency in comparison with several conventional CB_1_ receptor blockers but act differently to AM251 by the reverse modulation of CB_1_ receptors [56]. A recent study showed that chelerythrine produces the sequential activation of muscarinic (M_3_) receptors and CB_1_ receptors which synergistically induce contractile effects of the bovine ciliary muscle by involving the activation of Rho-kinase and PKC [62]. Considering the CB selectivity these molecules may serve as a template for potent CB_1_ receptor blocking drugs of natural origin negatively regulating the ECS.

### 2.11. Curcumin

Curcumin, chemically known as diferuloylmethane, is a well-known polyphenol molecule and an active constituent of the dietary spice turmeric (*Curcuma longa*) used for dietary and medicinal purposes since centuries. Numerous studies demonstrate that curcumin regulates various signaling molecules including inflammatory molecules, cytokines and chemokines, adhesion molecules, transcription factors, enzymes, protein kinases, protein reductases, carrier proteins, cell survival proteins, cell-cycle regulatory proteins, drug resistance proteins, growth factors, receptors, DNA, RNA, and metal ions. Seely et al. [63] first showed that curcumin binds to CB_1_ receptors with nanomolar affinities and in micromolar affinities with CB_2_ receptors. Structurally, curcumin also shares structural motifs with some cannabinoid receptor ligands. Further, curcumin has been showed to cause sustained elevation of brain derived nerve growth factor and endocannabinoids in brain region-specific and dose-dependent manner similar to the conventional antidepressant amitriptyline [64]. However, pretreatment with AM4113, a CB_1_ receptor neutral antagonist, but not with SR144528, a CB_2_ receptor antagonist, prevents induction of brain derived nerve growth factors and suggests CB_1_ receptor mediated ECS as novel targets for curcumin. Recently, Witkin et al. [65] reported that curcumin did not potently alter GTP-*γ*-35S binding, which suggests its functional CB_1_ antagonist (*K*_*i*_ = 2080 nM). Further, curcumin did not prevent the hypothermic effects of the CP55,940 and the anti-immobility effects of curcumin did not occur in CB_1_ knockout (CB_1_^−/−^) mice. In a recent study, Zhang et al. [66] demonstrated the cannabinoid mediated antifibrotic activity of curcumin in liver fibrosis induced by carbon tetrachloride. Curcumin treatment upregulated CB_2_ receptors and downregulated CB_1_ receptors in hepatic stellate cells and modulated the expression of extracellular matrix (ECM) proteins. The abrogation of inhibition of curcumin effects on ECM expression revealed that inverse agonism/antagonism of CB_1_ receptors contributed to curcumin inhibition of ECM expression. Further,* in silico* studies showed its binding to CB_1_ receptors with two hydrogen bonds. In a very recent study, bisdemothoxycurcumin, a derivative of curcumin, has been showed to induce apoptosis in activated hepatic stellate stem cells by impairing cellular energetics and downregulating cytoprotective proteins, likely through a mechanism that involves CB_2_ receptors as evidenced by reversal of the BDMC-induced apoptosis with cotreatment of SR144528, a CB_2_ antagonist, and confirmed with genetic downregulation of the receptor using siCB_2_ receptors [67]. The studies conclude that the effects of curcumin in chronic liver disease are mediated by cannabinoid receptors and may offer therapeutic benefits in hepatic fibrosis. Integrated all together, cannabinoid mediated effects of curcumin and well established manifold properties of curcumin; it holds a strong propensity in diseases where ECS is dysregulated.

### 2.12. Haplosamate

Haplosamate derivatives are first naturally derived cannabinomimetic compound belonging to steroid family representing a new chemical class of cannabinoid receptor ligands. It is a group of steroids including haplosamate A and haplosamate B [68, 69]. Haplosamate A is a C28 sterol containing seven oxygenated carbons and a rare six-member ether ring connecting C-16 and C-23 with a sulfate group at C-3 as well as a methyl phosphate at C-15. For the first time, it was isolated from a sponge,* Xestospongia* sp., and later on from other sponges such as Haplosclerida spp. and* Cribrochalina* spp. [68] and Indonesian marine sponge,* Dasychalina* spp. (family: Niphatidae). The isolated haplosamate compounds, haplosamate A and desulfohaplosamate, as well as semisynthetic derivatives were screened for the interaction and affinity to cannabinoid receptor. Haplosamate A and desulfohaplosamate exert opposite effects as haplosamate A showed significant affinity for CB_1_ receptor, whereas desulfohaplosamate showed higher affinity for CB_2_ receptor. The 7-monoacetylated derivative of haplosamate A exhibits affinity to both cannabinoid receptors in comparison with its parent compound. However, acetylation at C-4 or dialdehyde derivative showed the loss of affinity on both CB_1_ and CB_2_ receptors.

### 2.13. Euphol

*Euphol*, a tetracyclic triterpene alcohol, is the key constituent in the sap of* Euphorbia tirucalli* L. (family: Euphorbiaceae), a plant grown in Africa and South America, Brazil, and Amazonas. King et al. [70] first reported that euphol inhibits MAGL in a reversible and noncompetitive manner. The SAR studies reveal that euphol is a bioisoester of pristimerin and lacks the quinone methide group and is found devoid of CNS side effects in the tetrad tests, such as deficit locomotor, catalepsy, analgesia, and hypothermia, typical features of cannabinoids. Euphol showed potent immunomodulator and anti-inflammatory effects in animal models of ulcerative colitis and autoimmune encephalomyelitis where CB_2_ receptors play a vital role in pathogenesis [71]. The antihyperalgesic effect of euphol appears similar to the effects caused by ACEA, a CB_1_ receptor agonist, and JWH-133, a CB_2_ receptor agonist. The reversal of the antinociceptive effects of euphol on pretreatment with CB_1_ antagonist AM251 or with CB_2_ selective antagonist AM630 showed CB_1_ and CB_2_ receptor dependent mechanisms. Euphol was found efficacious in preventing the neuropathic behavior mediated through the modulation of both CB_1_ and CB_2_ receptors. These findings suggest that euphol has excellent potential for use in neuropathic pain and persistent inflammation owing its ability to interact with ECS and is devoid of the CNS adverse effects even at high doses.

### 2.14. Falcarinol

Falcarinol is a C17-polyacetylene compound with two carbon-carbon triple bonds and two double bonds and possesses a reactive polyyne structure and is found predominantly in carrot, celery, fennel, parsnip, and Gamisans, members of Araliaceae and Apiaceae family. It is a phytoalexin also known as panaxynol and isolated for the first time from* Panax ginseng*. It showed to bind with both cannabinoid receptors nonselectively but selectively alkylates the CB_1_ receptors and induces CB_1_ receptor mediated functional signals by covalent and irreversible interaction with the CB_1_ receptors (*K*_*i*_ = 0.59 *μ*M) [72]. Though, falcarinol is not a functional ligand at CB_2_ receptor as it did not interfere with constitutive or forskolin-stimulated cAMP but appears as a weak partial agonist on CB_2_ receptor and acting through G_o_ signaling [72]. Falcarinol is unstable and upon exposure to sunlight causes the formation of secondary alcohol with the loss of binding affinity to the cannabinoid receptors. Thus, only freshly obtained falcarinol exerts significant cannabinoid receptor binding affinity. Recently, falcarinol showed inverse agonist/antagonism for the CB_1_ receptors in keratinocytes and causes expression of proallergic chemokines in keratinocytes, the effects similar to rimonabant. Furthermore, a structural analog of falcarinol, pontica epoxide, was found devoid of affinity either for cannabinoid or for opioid receptors [15].

### 2.15. 18*β*-Glycyrrhetinic Acid

18*β*-Glycyrrhetinic acid and its diastereomer 18*α*-GA are the triterpenoid saponins obtained from the roots of* Glycyrrhiza glabra* L., popularly known as licorice. It is generally used as a natural sweetener and flavoring additive in food and as traditional medicines owing to its antimicrobial, anticancer, and anti-inflammatory properties. The inhibitory activities of licorice extract in hCB_1_ receptor-expressing Chem-1 cells showed a dose-dependent decrease in intracellular Ca^2+^ levels (IC_50_ = 1.96 ± 0.05 *μ*M) [73]. Other active constituents of licorice like liquirtin, glabridin, and 18*α*-glycyrrhetinic acid also exhibited inhibitory activity against Ca^2+^ flux induced by AEA, whereas 18*β*-glycyrrhetinic acid showed stronger potency evidenced by more than 90% inhibition in responses to CB_1_ receptor agonist. The 18*β*-glycyrrhetinic acid was also found to regulate CB_1_ receptors implicated in antiadipogenesis responses in 3T3-L1 cells and exerts antiobesity effects by correcting lipid dysregulation, body weight gain in diet-induced obese animals [73]. Further, it also alleviated effects of AEA, a CB_1_ receptor agonist, and suppressed adipocyte differentiation in 3T3-L1 cells by downregulating the AEA-induced MAPK activation and expression of adipogenic genes including C/EBP-*α* and PPAR-*γ*. The 18*β*-glycyrrhetinic acid in licorice extract appears to be an active constituent possessing CB_1_ receptor downregulatory effect and confers therapeutic effects against obesity.

### 2.16. Guineensine

Guineensine possesses potent cytotoxic, insect repellents, anti-inflammatory, insecticidal, and antifeedant activities from black pepper,* Piper nigrum* (family: Piperaceae). It appears as a potent novel inhibitor (EC_50_ = 290 nM) of cellular uptake of the AEA and 2-AG [74, 75] in nanomolar range. Though, guineensine did not inhibit the enzyme FAAH or enzyme MAGL or interact with cannabinoid receptors or fatty acid binding protein 5 (FABP5), a major cytoplasmic AEA carrier, or serine hydrolases. The SAR studies suggest the significance of alkyl chain length interconnecting the pharmacophoric isobutylamide and benzodioxol moieties for AEA cellular uptake inhibition. Studies have shown cannabimimetic effects such as catalepsy, hypothermia, reduced locomotion, analgesia, and blockade of the effects by CB_1_ receptor antagonist, rimonabant (SR141716A) in animals. Other common constituents of black pepper, piperine, dose-dependently reduce intestinal fluid accumulation induced by castor oil and pretreatment with SR141716A; a CB_1_ receptor antagonist showed that the effects were not dependent on cannabinoid receptors [76]. Similarly, Izzo et al. [77] studied the effect of capsaicin, piperine, and anandamide on upper gastrointestinal motility in mice and showed the inhibitory effect of anandamide but not piperine using a noneffective dose of SR141716A, a CB_1_ receptor antagonist. Piperine appears to reduce upper gastrointestinal motility independent of CB_1_ receptors. Guineensine appears as a novel plant derived compound which inhibits endocannabinoid uptake independent of FAAH [74, 75]. Thus, the scaffold of guineensine could be useful in finding future tools for ECS transport and modulatory mechanism in therapeutics.

### 2.17. Hydroxyeicosatetraenoic Acid (HETE) and Hydroxyl-Anandamide (HAEA)

The oxylipin, 3-hydroxyarachidonic acid (3(*R*)-HETE), is an intermediate of the *β*-oxidation of arachidonic acid and plays an important biological role in the life cycle of fungi. The fungal pathogen* Candida albicans* transforms arachidonic acid into 3(*R*)-HETE. It has been showed that* Diposascopsis uninucleata* converts AEA into 3-HAEA and established an enantiodivergent synthesis to study its pharmacological activity [78]. The affinity of AEA, 3(*R*)-HAEA, and 3(*S*)-HAEA for CB_1_ receptors was 0.02 ± 0.015 *μ*M, 1.85 ± 0.275 *μ*M, and 1.46 ± 0.33 and for CB_2_ receptors was 0.11 ± 0.025 *μ*M, 6.43 ± 0.7710 *μ*M, and 4.85 ± 0.38 *μ*M, respectively. Thus, yeasts producing 3(*R*)-HETE convert AEA released by the host cells at the site of infection into 3(*R*)-HAEA which leads to the inflammatory and algogenic responses associated with fungal diseases. Both the enantiomers of 3-HAEA exhibited similar affinity for hCB_1_ and hCB_2_ receptors but significantly (approximately 70–90-fold and approximately 40–60-fold) lower affinity than the parent compound AEA. Further, studies are needed in order to utilize these compounds in drug discovery through biotransformation.

### 2.18. Magnolol

Magnolol, a biphenyl neolignan from* Magnolia officinalis*, was used popularly in traditional Chinese medicine for insomnia, anxiety, and allergic diseases. Rempel et al. [79] examined the extract and biphenyls honokiol, magnolol, 8,9-dihydromagnolol, tetrahydromagnolol, and trans-isomagnolol for its cannabinoid affinity and activity. The study showed that magnolol behaved as partial agonist for CB_2_ receptor, while honokiol was less potent but showed full agonistic activity at CB_1_ and antagonistic properties at CB_2_ receptor. However, further studies showed no inhibition activity for FAAH and MAGL in rat brain preparations. Thus magnolol showed partial agonist affinity at both CB receptor subtypes, while tetrahydromagnolol showed higher affinity for CB_2_ receptor and antagonist at GPR55, a CB-related orphan receptor in *β*-arrestin translocation assays.

Fuchs et al. [80] synthesized analogs of magnolol and investigated affinity at hCB_1_/CB_2_ receptors using CP55,940 radioligand studies and also examined SAR of these analogs with variations of alkyl chains and phenolic groups which may improve the potency. The study showed that methylation of phenolic hydroxyl group abolishes the preference of magnolol analogs for CB_2_ receptors; however depending on which of the two phenolic groups was methylated the resulting compounds exhibited an enhanced affinity to CB_1_ receptors. Full agonism on CB_1_ and CB_2_ receptors was observed following methylation of the hydroxyl group in the* para*-position to the propyl residue for derivatives. But methylation of the hydroxyl group in the* para*-position of the hexyl residues results in CB_1_ antagonist and partial CB_2_ receptors agonist activity, emphasizing the importance of the free phenolic hydroxyl group for high intrinsic activity. Further, activity of new analogs at G_i_-coupled CB_1_ and CB_2_ receptor subtypes on forskolin-stimulated adenylate cyclase activity in cAMP accumulation assays confirmed that potency and efficacy of magnolol can be easily altered by methylation of one of the phenolic hydroxyl groups and depending on the position of the methoxy group, full agonism on both receptors with antagonist activity at CB_1_ and partial agonist activity at CB_2_ receptors can be achieved. Magnolol also exhibited dual agonism of RXR*α* and PPAR*β*/*γ* and appears as an important agent to target this heterodimer [81]. The manipulation of the biphenyl scaffold appears as a putative pharmacophore for the further development of novel CB receptor ligands.

### 2.19. Malyngamides

Malyngamides are the fatty acid amide compounds abundantly found in marine cyanobacterial metabolites from* Lyngbya* spp. Till date, more than 30 malyngamide analogues have been isolated and screened for their cannabinoid affinity and activity. Among numerous analogues, malyngamide B appeared to bind to both CB_1_ and CB_2_ receptors, with moderate potencies as agonist. Further tests reveal its anti-inflammatory properties like cannabimimetic compounds and it was found to inhibit NO production with an IC_50_ of 6.2 *μ*M without affecting cellular viability up to 25 *μ*M. It appears devoid of inhibitory activity on FAAH, which catalyzes anandamide hydrolysis and terminates anandamide signaling [82].

### 2.20. Rutin

Rutin is a flavonoid from* Saussurea involucrata* also known as snow lotus, in different regions of China. The cannabinoid mediated antidepressant activity of rutin shown in mice models employing weight-loaded forced swim test. Rutin treatment showed upregulation of CB_1_ receptors in mouse brain tissue demonstrating antifatigue activity and CB_1_ receptor-interacting proteins. Further, in brain tissues, an increase in expression of peroxisome proliferator-activated receptor-*α* coactivator (PGC-1*α*) and sirtuin 1 (SIRT1) was also demonstrated [83]. Integrating together the cannabinoid, PPAR-*γ*, and opioid receptor activities, rutin may be a potential multitargeted polypharmacological agent in prevention and treatment of diseases involving dysregulation of PPAR and ECS.

### 2.21. Serinolamides

Serinolamides are fatty acid amides found in a marine cyanobacterium,* Lyngbya* spp., collected from Piti Bomb Holes from Guam. Among the isolated compounds, the analogue serinolamide A isolated from the marine cyanobacterium,* Lyngbya majuscula*,* Oscillatoria* spp., showed structural similarity to the endocannabinoids anandamide and 2-AG. Serinolamide A showed binding affinity to the human cannabinoid receptors and found 5-fold more selective agonist activity for the CB_1_ receptors with moderate binding affinity [84], whereas serinolamide B appeared to inhibit forskolin-stimulated cAMP accumulation mediating both CB_1_ and CB_2_ receptors with moderate potencies along with more CB_2_ receptor selectivity in binding as well as functional assays. However, serinolamide B showed an opposite trend in binding affinities compared to serinolamide A, where it exhibited a moderate affinity and higher selectivity for CB_2_ (*K*_*i*_ = 5.2 *μ*M) over CB_1_ receptor (*K*_*i*_ = 16.4 *μ*M) [82]. Serinolamide B like other cannabimimetic compounds exerted anti-inflammatory effects in lipopolysaccharide- (LPS-) induced murine macrophages RAW 264.7 with an IC_50_ > 25 *μ*M. The observations indicate that presence of a secondary amide versus a tertiary amide is not a major element for specific receptor selectivity. Though, the compounds represent a novel scaffold from a marine organism for the development of cannabinoid modulators.

### 2.22. Methylhonokiol

4′-O-Methylhonokiol is a polyphenolic compound isolated from* Magnolia grandiflora* L., a tree growing in Northern Mexico and the USA. Schuehly et al. [85] first reported methylhonokiol as a potent agonist on CB_2_ receptors, triggering a novel type of heteroactive signaling in the radioligand displacement assays in HEK293 cells. In an* in vitro* study, methylhonokiol only showed ligand binding interactions with CB_2_ receptors but no effects on GPR55 and CB_1_ receptors. It also acts both as inverse agonist and as agonist dependent on the specific signal pathways. A prominent effect of methylhonokiol observed is inhibition of macrophage migration induced by 2-AG, even though it shows anti-inflammatory properties similar to 2-AG and other endocannabinoids [86].

Based on the reports that orally administered 4′-O-methylhonokiol prevents amyloidogenesis and progression of Alzheimer's disease by inhibiting neuroinflammation in mouse model of Alzheimer's disease [87], authors also suggested that 4′-O-methylhonokiol exerts its beneficial effects by modulation of CB_2_ receptors significantly expressed in astrocytes and microglia. Its structural similarity with HU308, a synthetic CB_2_ receptor-selective agonist, has been shown to inhibit osteoclastogenesis and be useful as bone resorption inhibitors support its cannabinoid property [88]. Overall, with activities such as GABAergic, PPAR-*γ*, and AChE modulatory, methylhonokiol seems to be a novel agent to target CB_2_ receptors in treatment of osteoarthritis, Alzheimer's disease, neuroinflammation, neuropathic pain, and chronic bowel disease.

### 2.23. Miconioside

Miconioside compounds are flavanone glycoside isolated from the methanolic extract of the stems of* Miconia prasina* growing in tropical and subtropical regions of the Americas. These compounds include miconiosides B and C which showed their affinity to bind with CB_1_ and CB_2_ receptors. They showed weak inhibition for CB_2_ receptors, but no activity on CB_1_ receptors in radioligand binding studies [89].

### 2.24. Pristimerin

Pristimerin is a natural quinone methide triterpenoid isolated from the* Celastrus* and* Maytenus* spp. exhibiting anti-inflammatory, antioxidant, chemoprotective, and antimalarial activity. Pristimerin exhibits reversible inhibition of MAGL [70] as the quinone methide group to react with cysteine residues of proteins to form covalent adducts [90] and this was confirmed by using a rapid dilution assay. The molecular docking studies showed that lipophilic portion of the molecule lies on a pocket located within the lid domain of MAGL and its 3-hydroxyl group [70]. The binding of pristimerin to MAGL strengthens by the formation of a polar interaction with a regulatory cysteine, possibly Cys^208^. Chicca et al. [34] also showed that pristimerin and JZL184 both produce potent inhibition of MAGL activity. Pristimerin produced inhibition of [^3^H]-glycerol formation and accumulation of intracellular [^3^H]2-AG and was found less potent than *β*-amyrin, another MAGL inhibitor. Based on the* in vitro* and* in vivo* studies, it has been concluded that pristimerin inhibits MAGL in a rapid, reversible, and noncompetitive manner.

### 2.25. Resveratrol

Resveratrol is a stilbenoid compound isolated from fruits and plants and widely studied for its pharmacological properties. Recently, the uncharacterized trans-resveratrol receptor has shown to share many characteristics with cannabinoid receptors. The affinity of trans-arachidins, trans-resveratrol, and trans-piceatannol for CB_1_ and CB_2_ receptors was investigated in CHO cells expressing cannabinoid receptors and it was found that trans-resveratrol and all analogs bind to CB_1_ receptors, whereas isoprenylated trans-resveratrol derivatives tA1 and tA3 bind to CB_2_ receptors [63]. The study showed affinity of trans-resveratrol and trans-piceatannol for CB_2_ receptors is 5- to 10-fold lower than that observed for CB_1_ receptors. All compounds except for tA3 exhibit approximately 2- to 10-fold selectively for binding to CB_1_ receptors relative to CB_2_ receptors. In molecular docking, trans-arachidins, trans-resveratrol, trans-piceatannol, and their glucuronidated metabolites bind with CB_2_ receptors while isoprenylated analogs tA1 and tA3 bind with both CB_1_ and CB_2_ receptors. Trans-resveratrol and Trans-picetamol also bind to mCB_1_ receptors; however they lack affinity for hCB_2_ receptors. The docking studies showed that prenylated stilbenoids trans-arachidins 1 and 3, the more lipophilic isoprenylated analogs of trans-resveratrol and trans-piceatannol, may be preferable alternatives to trans-resveratrol due to increased bioavailability via slowed metabolism. Both parent and isoprenylated compounds bind to CB_1_ receptors and were confirmed by the antagonistic actions produced by CB_1_ receptor agonists. However, the analogs possess an isoprenyl group, trans-arachidin 1 and trans-arachidin 3, showed affinity for CB_2_ receptors, and were further confirmed by molecular docking [91]. Though, resveratrol has been well investigated in numerous experimental and clinical studies; however the cannabinoid mediated pharmacological effects need to be ascertained.

### 2.26. Resorcylic Acid Lactones

Resorcylic acid lactones neocosmosin A, neocosmosin B, neocosmosin C, monocillin IV, monocillin II, and radicicol are obtained from ethyl acetate extracts of* Neocosmospora* spp. The extracts as well as the compounds were found to exhibit moderate affinity with opioid receptor and cannabinoid receptors in a high throughput screen employing a receptor binding assay. Among these compounds, neocosmosin B, monocillin II, and radicicol showed a binding affinity for CB_1_ receptors using CP55,940 as standard. However, compounds, neocosmosin A, neocosmosin B, neocosmosin C, monocillin II, and radicicol, exhibited binding affinity to CB_2_ receptors with respect to CP55,940 as standard. Neocosmosin C, monocillin II, and radicicol also showed good affinity for binding with the human opioid receptors [92, 93]. These findings are implicated in neuropathic pain and neuroinflammatory disorders where opioid and cannabinoid systems are dysregulated.

### 2.27. Salvinorin A

Salvinorin A, a trans-neoclerodane diterpenoid, is the principal constituent of* Salvia divinorum*, a plant used in Mexico for spiritual and medical purposes. It possesses psychotropic activity that resembles with the structure and mode of action of typical hallucinogens. The radioligand displacement studies show salvinorin A as a potent, selective, and full agonist on *κ*-opioid receptors [94–96], but not *μ*- or *δ*-opioid receptors. Other studies have shown that salvinorin A possesses ECS mediated activity and interaction with *κ*-opioid in rats and Zebra fish models [94, 96–101]. It provides a new lead compound for developing antiallodynic agents via opioid and CB_1_ receptors activation. Fichna et al. [102] demonstrated that salvinorin A impedes gastrointestinal motility and ion transport, mediated by *κ*-opioid receptors in mice. Further, it significantly attenuated chemical-induced colitis in mice and the antinociceptive action was blocked by opioid and CB_1_ receptor antagonists. Salvinorin A also slows colonic motility* in vitro* and* in vivo* and alters neurogenic ion transport [103, 104]. Further, Fichna et al. [105] reported the inhibitory effects of salvinorin A on endotoxin-induced ileal hypercontractility in mouse stomach mediated by opioid receptors and cannabinoid receptors. The inhibitory effect of salvinorin A on motility demonstrates functional interaction between CB_1_ and *κ*-opioid receptors in the inflamed gut but in normal control animals [98].

Further, Aviello et al. [100] reported that salvinorin A reduced inflammation and pain in animal models of LPS- and carrageenan-induced paw edema as well as formalin-induced inflammatory pain. The actions were found mediated by the *κ*-opioid receptors and CB_1_ receptors-dependent anti-inflammatory actions on macrophages and in experimental animals. A study evaluated salvinorin A in a set of* in vitro* and* in vivo* tests and demonstrated that salvinorin A did not bind or activate CB_1_ receptors but effects are mediated by its activation of *κ*-opioid receptors [96]. Braida et al. [95] reported the anxiolytic- and antidepressant-like effects of salvinorin A which are mediated by both *κ*-opioid and CB_1_ receptors. In addition to a weak affinity for CB_1_ receptors, it also reduced FAAH activity in amygdale. Based on the cannabinoid and opioid modulatory activity, salvinorin A or its synthetic or semisynthetic derivatives could be useful in the treatment of lower gastrointestinal disorders because inflammation in the intestine upregulates cannabinoid receptors and endogenous cannabinoids.

### 2.28. *γ*-Sanshool

*γ*-Sanshool is an alkylamide compound isolated from* Zanthoxylum clava-herculis* L. (family: Rutaceae) also known as pepperwood, native to the southeastern United States. Dossou et al. [106] have shown its CB_2_ receptor activity. Subsequently, a novel plate-based assay was developed in order to determine both CB_1_ and CB_2_ receptors antagonist and agonist activity and the ligand effect on internalization of the CB_1_/CB_2_ receptors in different extracts of the plant genus* Zanthoxylum* [106].

Later, it was found that *γ*-Sanshool isolated from* Zanthoxylum bungeanum* shows potent agonism on the CB_2_ receptor and antagonism on CB_1_ receptors. In addition to its interactions with CB_1_ and CB_2_ receptors, it showed antagonist activity at the follicle stimulating hormone receptor (68%) and at the prolactin-releasing hormone receptor (52%). These findings reveal that, given the role of cannabinoid receptors in diabetes pathophysiology, *γ*-Sanshool with a dual function on CB_1_ receptors inhibition in combination with CB_2_ activation may be useful in the treatment of diabetes.

### 2.29. Sciadonic Acid

Sciadonic acid is obtained from the seeds of a coniferous plant,* Sciadopitys verticillata* (umbrella pine) in Japan. Sciadonic acid structurally resembles with 2-AG, the endogenous natural ligand for the cannabinoid receptor. Nakane et al. [107] showed that sciadonic acid exhibits cannabimimetic activity by inducing rise of intracellular Ca^2+^ levels in neuroblastomaxglioma hybrid cells (NG108-15) expressing CB_1_ receptors. This was the first study showing the occurrence of a cannabimimetic monoacylglycerol in higher plants exhibiting CB_1_ receptor dependent mechanism.

### 2.30. Semiplenamides

Semiplenamides (semiplenamide A to G) belong to a series of novel fatty acid amides similar to endocannabinoid, anandamide. These were isolated from marine blue green algae,* Lyngbya semiplena* collected from Papua New Guinea. Semiplenamides A, B, and G derivatives exhibited weak affinity for the CB_1_ receptors [108]. Additionally, semiplenamide A was found to be a moderate inhibitor of the anandamide membrane transporter thereby inhibiting anandamide breakdown. The results indicate that these compounds may appear as future cannabinoid specific drugs of natural origin.

### 2.31. Thujone

Thujone, a monoterpene ketone, is found in variable amounts in several food and medicinal plants such as* Juniperus* spp.,* Cedrus* spp. It has been regarded as a severe neurotoxicant causing exciting and convulsive effects in the CNS by inhibiting GABA_A_ receptors in a dose-dependent manner. It is known for its notoriety being an important component of the once-popular drink absinthe. Thujone possesses psychoactivity similar to cannabinoids but does not mimic cannabinoids in inhibiting the synaptosomal enzyme [109].

Meschler and Howlett [110] investigated the affinity of thujone for the brain CB_1_ receptor in radioligand assay and found that thujone affinity with the CB_2_ receptor is approximately similar to the CB_1_ receptor. In bioassays and forskolin-stimulated adenylate cyclase assays, thujone did not show any activity on CB_1_ receptor. Thujone treatment in rats exhibited different behavioral characteristics, the open-field test for locomotor activity, the ring-stand test for immobility (catalepsy), and hot-plate test for antinociception comparable with a potent cannabinoid agonist, levonantradol. Though, thujone was found devoid of stimulatory activity on brain cannabinoid receptors and does not elicit cannabimimetic behavioral effects in animals at physiologically relevant doses.

### 2.32. Voacamine and Analogues

Voacamine, 3,6-oxidovoacangine, and 5-hydroxy-3,6-oxidovoacangine are the indole alkaloids isolated from methanolic extract of root bark of* Voacanga africana*, a tropical African tree. Several compounds have been isolated and screened for the cannabinoid activity in Aequorin/GPCR cell-based Ca^2+^ functional assay using CP55,940 or rimonabant as a positive control for cannabinoid receptors ligands [111]. These compounds exhibited potent CB_1_ receptor antagonist activity in a concentration-dependent manner compared to rimonabant, whereas the other coexisting alkaloids, such as voacangine, vobasine, and tabersonine, fail to exhibit any CB receptor mediated activity. This was the first study showing that naturally occurring alkaloids are also source of CB_1_ receptor antagonists and this could be further evaluated for cannabimimetic activity and potential therapeutic benefits.

### 2.33. Yangonin

Yangonin is a kavalactone extracted from* Piper methysticum* Forster, popularly known as Kava, and cultivated in the South Pacific Island Countries. Several compounds, known as kavalactones, are isolated and the most common are kavain, 7,8-dihydrokavain, methysticin, 7,8-dihydromethysticin, yangonin, and desmethoxyyangonin. Ligresti et al. [112] examined their CB receptor binding affinity and inhibitory activity on endocannabinoid metabolizing enzymes, FAAH and MAGL involved in endocannabinoid degradation. Only yangonin emerged as the most interesting compound as evidenced by the binding affinity to the CB_1_ receptor (*K*_*i*_ = 0.72 *μ*M). However, all other compounds were found inactive in inhibiting activities of FAAH and MAGL enzymes.

The study also reported that 250–1250 mg yangonin, which is 10% of the total kavalactone-content taken orally, may provide sufficient serum concentrations of yangonin to affect CB_1_ receptors in the CNS. The authors suggested that yangonin which possesses an extensive conjugated double bond system bears a little structural resemblance to the phytocannabinoids. The kavalactones may also be a target for GABA and BZDs, voltage gated Na^+^/Ca^2+^ channels, monoamine uptake, and arachidonate cascade which may synergize and contribute to the psychopharmacological profile of the Kava. 

*Miscellaneous Compounds Isolated from Nature*. Desmodianone derivatives, desmodianones D and E and 6-methyltetrapterol A, are isoflavonoids isolated from* Desmodium canum*. It is known for soil preserving property and used as forage with some application in traditional medicine. These isoflavonoids possess cannabinoid-like moieties; however no further reports on their cannabimimetic or cannabinoid modulatory activity are available in the literature [113]. Isoperrottetin A, a bibenzyl compound along with several bisbenzyls, prenyl bibenzyls, and sesquiterpenoids, has been isolated from the ether extract of the liverwort,* Radula perrottetii*. All these compounds are known to structurally consist of cannabinoid moiety; however there is no report available on their ECS modulating property [114].

Leucettamols are the bifunctionalized sphingoid-like compounds obtained from a marine sponge,* Leucetta* sp. In preliminary studies, they appear inactive on CB_1_, CB_2_, and TRPV1 receptors. Soderstrom et al. [115] also extracted numerous endocannabinoid-like purified unsaturated fatty acids from green algae (Chlorophyta), the brown alga* Laminaria angustata*, and the sponge* Mycale micracanthoxea*. The authors did not find endocannabinoid compound from* L. majuscula*. Also, AEA has been detected in dietary chocolate and cocoa obtained from* Theobroma cacao*, a popular plant [116]. Recently, in a study, several compounds such as sinostrobin, naringenin 7,4′-dimethyl ether, 2′,6′-dihydroxy-4′-methoxychalcone, 4-methoxy-6-(2-phenylethenyl)-2H-pyran-2-one, naringenin 7-methyl ether, and 3,5-heptanediol, 1,7-diphenyl are isolated from the dichloromethane extract of* Renealmia alpinia* subjected to either opioid or cannabinoid receptors* in vitro* binding affinity assays. Though, the plants show antinociceptive and analgesic effect in the* in vivo* model but the constituents and plant failed to show affinity to cannabinoid receptors [117]. The compound isolated from the soil microfungus,* Eupenicillium parvum*, showed selective *μ*-opioid receptor and CB_1_ receptor binding affinities,* in vitro* binding assays [118]. These findings provide insight into the potential therapeutic utility of this class of compounds.

## 3. Medicinal Plants Modulating Cannabinoid Receptors and Metabolizing Enzymes

In the last few years, several medicinal plants have been reported to modulate the ECS activity by inhibiting or activating the cannabinoid receptors and the endocannabinoid metabolizing enzymes [22]. The plants have been reported to interact with cannabinoid receptors directly or indirectly in experimental studies designed to evaluate the pharmacological properties and therapeutic benefits using pharmacological challenge of CB receptor agonists and antagonists or utilizing the CB_1_/CB_2_ receptor knockout mice [6, 19, 22]. Several medicinal plants other than cannabis have been shown to alter the ECS signaling pathways and exhibit cannabimimetic effects and put forward their potential therapeutic and dietary application [6, 16–19]. The therapeutic and pharmacological activities presented by these plants involving cannabinoid mediated activity are present in Table 4.

In the modern era of medicine medicinal plants and phytochemicals derived from plants continue to play an important role in drug discovery and development [6, 16, 17, 41]. The plants have become the key resource for bioactive agents and played a vital role in the search of lead compounds for novel drug discovery and development. The isolated bioactive agents and their synthetic or semisynthetic analogs can be developed into promising drug candidates by the processes of highly efficient bioactivity-directed fractionation and isolation, following analog synthesis using modern medicinal chemistry-based molecular modifications. The next paragraphs focus on medicinal plants other than cannabis which have been reported to interact with the molecular components of ECS and are detailed below. The bioactive constituents of such plants display a rich source for the discovery of novel cannabinoid compounds with potential for pharmacological applications and drug development. Besides the small molecules, secondary metabolites also play an important role in search of novel compounds.

### 3.1. *Corydalis yanhusuo*

*Corydalis yanhusuo* (family: Papaveraceae) is one of the traditional Chinese medicines used as sedative, hypnotic, and pain killer possessing a number of potent alkaloids. The CB_1_ receptor mediated effect of* Corydalis yanhusuo* was tested in an animal model of trigeminal neuralgia pain induced in rats by chronic constriction injury of the infraorbital branch of the trigeminal nerve [119].* Corydalis* binds to CB_1_ receptors and exerts antinociceptive effect in animal models of inflammation and pain. In addition, tetrahydropalmatine [127] an active component isolated from* Corydalis* has shown to improve anxiolysis and decreased motor movements, independent of the GABA_A_ receptors [127]. The analgesic and anti-inflammatory effect mediated by CB_1_ receptors along with anxiolytic activity is an advantage over synthetic CB_1_ receptor modulators [127].

### 3.2. *Echinacea purpurea*

*Echinacea purpurea* is commonly used worldwide for the prevention and treatment of common cold, cough, bronchitis, influenza, and allergic respiratory diseases. It has been shown to exert antioxidant, anti-inflammatory, and immunostimulatory properties owing to the chemical constituents, alkamides, and ketoalkenes/alkynes. The alkamides were the first compounds identified in plants besides cannabis to possess cannabimimetic properties on both the cannabinoid CB_1_ and CB_2_ receptors, revealing their structural similarity to the endogenous cannabinoid ligand anandamide [20, 128]. The extract of* Echinacea* roots was studied in ^[35S]^GTPcS-binding experiments on rat brain membrane preparations using arachidonyl-20-chloroethylamide (ACEA), a full agonist ligand at the CB_1_ receptor [128]. Among the isolated compounds, some displayed partial agonist property while others exhibit inverse agonist effects to CB_1_ receptor. Despite their relatively low efficacy at the cannabinoid receptors, the compounds behave as inverse agonist was capable of inhibiting the full agonist effect of ACEA. The compounds showed partial agonistic property that also significantly increased the G-protein-stimulatory action of ACEA. The SAR studies showed an exchange of isobutylamide moiety (inverse agonist activity) of the molecule for 2-methylbutylamide (partial agonist activity).

The CB_2_ receptor activity of alkamides, demonstrated by binding assays, is believed to be the most probable mechanism of action of alkamides as immunomodulator agents isolated from* Echinacea* [21, 25, 27, 129]. The interaction between anxiety and cannabinoids is known to be complex and activation of the CB_1_ receptors by endogenous ligands was believed to play a role in the control of anxiety [128]. The dry and fresh herb of* Echinacea* provides a different yield of alkamides [29, 30]. All together the studies convincingly suggest that* Echinacea* could provide scaffolds for future CB_2_ ligands in drug discovery and development.

### 3.3. *Linum usitatissimum*

*Linum usitatissimum* (family: Linaceae), also known as flax, is considered a distinct source of fibers and oil for industrial and medicinal application. The transgenic plants are generated in order to enhance the production of phenylpropanoids; a class of new terpenoid has shown to possess health-beneficial properties. The plant has shown to alter the expression of genes involved in inflammatory processes in mouse and human fibroblasts and activates the gene expression of CB_2_ receptor [120]. The findings reveal that flax can be a source of cannabinoid-like compounds which may influence the immunological responses and aid in designing the fabric for wound dressing with putative anti-inflammatory properties [120].

### 3.4. *Melilotus suaveolens*

*Melilotus suaveolens* Ledeb. (family: Leguminosae), a traditional Tibetan medicine, is also known as wild alfalfa or “cold-tasting” annual or biennial herb. It has been reported to contain compounds such as coumarin, flavonoids, phenolic acids, steroids, and triterpenes. It has been found effective in inflammation, pain, and antimicrobial activity. The cannabinoid mediated anti-inflammatory activity of* M. suaveolens* has been demonstrated in a rat cecal ligation and puncture- (CLP-) induced animal model of acute lung injury representing sepsis in human [121]. It has shown to upregulate the CB_2_ expression in peripheral blood mononuclear cells, reduce the number of neutrophils, lymphocytes, and total cells, and inhibit the induction of proinflammatory cytokines and transcription factors, NF-*κβ*65. The CB_2_ expression was shown to be correlated negatively with NF-*κβ* mRNA and supported by a significant reduction in CLP-induced lung inflammation. These findings suggest that* M. suaveolens* may have therapeutic potential in the treatment of CLP-induced acute lung injury.

### 3.5. *Morinda citrifolia*

*Morinda citrifolia* L. (family: Rubiaceae), also known as Noni, has been used by Polynesians for over 2000 years for numerous diseases. The advent of Tahitian Noni Juice generated interest in medicine for its possible beneficial effects on human health and well-being. Almost all parts of the plants are used medicinally in treating a variety of ailments. Palu et al. [122] showed the binding affinities of Noni samples (Tahitian Noni Juice and Noni fruit juice concentrates) for CB_1_ and CB_2_ receptors in CHO-K1 cells expressing hCB receptors using WIN-55,212-2, a nonspecific ligand, and* in vivo* in mice. Both juices were found to activate CB_2_ receptor but inhibit CB_1_ receptors. Tahitian Noni Juice produced inhibition of CP55,940 for CB_1_ receptors and enhancement for CB_2_ receptors. Noni fruit juice concentrate caused stimulation of [^3^H]WIN-55,212-2 binding. At different concentrations the CB_1_ receptor was inhibited whereas CB_2_ receptor showed stimulations at the same concentrations.

The binding activity of Tahitian Noni Juice for CB_1_ receptors was similar at each concentration, even at fivefold increased concentration. However, at both concentrations a remarkable selectivity for CB_2_ binding/activation was observed for CB_2_ receptor [122]. In mice orally administered Tahitian Noni Juice decreased IL-4 and increased IFN-*γ* suggesting that Noni juice favorably alters the immune system and exhibits immunomodulatory effects by activating the CB_2_ receptors which are involved in the immune regulation. The dual activity of Noni juice as CB_1_ receptor inhibitor and CB_2_ receptor activator has potential benefits in inflammation and immunomodulation.

### 3.6. *Nelumbo nucifera*

*Nelumbo nucifera* Gaertn. (family: Nymphaeaceae), also known as a sacred lotus, is widely distributed across the world and used as food and medicine. The seeds, rhizomes, leaves, flowers, and roots of the plant have been reported to contain megastigmanes including eudesmane sesquiterpenes, nelumnucifosides A and B, alkaloids such as roemerine, nuciferine, nornuciferine, nelumboside, anonaine, 5-methoxy-6-hydroxyaporphine, liensinine, asimilobine, and flavonoids. The cannabinoid activity of both methanol and aqueous extracts of* N. nucifera* was studied in measuring inhibition of CP55,940 elicited CB_2_ activity in the G_i_/G_o_-coupled CHO-K1 cell line [123]. The methanolic extract showed antagonism against CP55,940 activity towards CB_2_ receptor, whereas water extract was found inactive. A potent antagonist activity towards CP55,940 activated CB_2_ receptor with an IC_50_ value of ~62.3 nM was demonstrated by AM630. The study indicated that* N. nucifera* petal extract possesses potential benefits in metabolic disorders mediated by antagonistic effect on CB_2_ receptors.

### 3.7. *Olea purpurea*

*Olea europaea* (family: Oleaceae), a traditional tree of the Mediterranean basin, is the source of olive oil. The effects of olive oil and its phenolic constituents on gene expression in ECS have been studied in human colon cancer cells (Caco-2). A selective and transient upregulation of CNR1 gene-encoding for CB_1_ receptor was induced by exposure of Caco-2 cells to the oil. However, the other ECS components such as CB_2_, GPR55, and TRPV1 receptors and endocannabinoid metabolizing enzymes, NAPE-PLD, DAGL, FAAH, and MAGL, remained unaffected [130].

Further, dietary oil supplementation was found to increase the expression of CB_1_ in the colon of rats. Following oil supplementation, the methylation of Cnr1 promoter, miR23a, and miR-301a, previously shown to be involved in the pathogenesis of colorectal cancer, was predicted to target CB_1_ mRNA and appears reduced. In another study, the phenolic compounds of olive oil were developed to allow the preparation of unsaturated derivatives altered food intake in rats owing to their molecular similarity with CB_1_ endogenous ligands and PPAR-*α* as potential targets [124]. Taken together, the findings demonstrate modulation of CB_1_ by olive oil or its phenolic compounds and may provide a new therapeutic avenue for prevention and treatment of cancer and obesity.

### 3.8. *Rubus coreanus* Miquel

*Rubus coreanus* Miquel (family: Rosaceae), also known as Korean black blackberry, is known for its benefits in liver and kidney diseases, spermatorrhoea, prostate, and urinary diseases. It is known to contain tannins such as sanguiin H-4 and sanguiin H-6, flavonoids such as 3,4-dihydroxybenzoic acid, nigaichigoside F1, nigaichigoside F2, and coreanoside F1, a dimeric triterpene glycosyl ester, and anthocyanins. Its supplementation has shown to enhance antioxidant capacity in men [131]. The cannabinoid receptors mediated activity of* Rubus coreanus* has been shown in osteoporosis and occurs with N-methyl-N-nitrosourea- (MNU-) induced prostatic hyperplasia in aged rats as well as diabetic osteoporosis rats [125] following streptozotocin or ovariectomization [132]. The upregulation of CB_1_ and CB_2_ receptors were increased in rats that were ovariectomized and treated with streptozotocin and* Rubus coreanus* but decreased in those treated with streptozotocin and* Rubus coreanus* alone. The study revealed that in postmenopausal diabetic and aged rats the antiosteoporotic effect is attributable to the CB receptor-related upregulation of osteoblastogenesis and inhibition of prostatic hyperplasia.* Rubus coreanus* rescued bone loss in diabetic and aged osteoporosis by simultaneous alteration of activation in osteoblasts and osteoclasts dependent on upregulation of the ECS. Though, the active component responsible for an effect is yet to be determined.

### 3.9. *Ruta graveolens*

*Ruta graveolens* L. (family: Rutaceae) is a plant of medicinal and culinary importance native to Mediterranean region of southern Europe and northern Africa and Balkans. The plant and phytochemicals isolated have shown to be effective in different types of skin diseases including psoriasis, vitiligo, and cutaneous lymphoma. The dichloromethane and methanol extracts of* Ruta graveolens* yielded several constituents and were subjected to* in silico* studies using hitting model for CB_2_ ligands consisting of the five selective agonists AM1241, GW405833, HU-308, JWH-133, and JWH-267 [126]. Of all the molecules subjected to parallel screening, rutamarin showed selective affinity to the CB_2_ receptor with a *K*_*i*_ of 2.64 ± 0.2 *μ*g/mL or 7.4 ± 0.6 *μ*M in radioligand displacement assay. The findings reveal that rutamarin may provide a novel scaffold for the discovery of CB_2_ specific ligands. 

*Miscellaneous Medicinal Plants.* Recently,* Withania somnifera* Dunal, a popular medicinal plant, possesses immunomodulator activity shown to prevent tolerance to the analgesic effect of morphine and suppress rebound hyperalgesia found devoid of affinity for cannabinoid receptors [133].* Hypericum perforatum* also known as St. John's wort is a popular plant remedy for depression that did not show cannabinoid property studied using the pharmacological challenge with several agonists and antagonists including SR141716A, CB_1_ receptor antagonist. However, naloxone significantly reduced the inhibitory effect of* Hypericum perforatum* on contractions induced by electrical field stimulation mediated by opioid receptors [134]. Yuliana et al. [135] evaluated the effects of several dietary spices in the antiobesity related bioactivity screening assays and found that nutmeg, mace, black pepper, and turmeric are capable of modulating the CB_1_ receptors. El-Alfy et al. [136] also showed that nutmeg extract showed a concentration-dependent inhibition for both FAAH and MAGL. Inhibition of endocannabinoid metabolizing enzymes by nutmeg extracts explains the cannabis-like effect exerted by nutmeg.

## 4. Concluding Remarks and Future Prospects

Compared to synthetic compounds, natural products are known to offer huge structural diversity and the availability of modern techniques for separation, structure elucidation, and screening and combinatorial synthesis will lead to revitalization of plant products as sources of novel drugs. In recent years, several new selective CB_1_ and CB_2_ receptor agents from natural products have been described. Though, several have been identified by these ligands* in vitro* and* in silico* studies. However, these molecules are used at micromolar concentrations in the* in vitro* studies and therefore may show affinity at both receptors. Therefore, additional controls are needed to be performed in order to ensure the selectivity, affinity, potency, and site of action of these molecules.

The* in vivo* characterization, pharmacokinetic considerations, and the cannabinoid mediated mechanism should be demonstrated for the pharmacological benefit and pharmaceutical development. Moreover, many of these ligands exert prominent CB receptor-independent pharmacological effects, such as activation of the opioid receptors, nicotinic acetylcholine receptors, G-protein-coupled receptor GPR55, peroxisome proliferator-activated receptor gamma, and the transient receptor potential vanilloid channels. The characterization of CB-dependent and CB-independent mechanisms could be further beneficial in developing the multitargeted polypharmacological compound for diseases which involve multiple mechanisms particularly the neurodegenerative and neuropsychiatric diseases where endocannabinoid system dysregulation plays a critical role. Based on current knowledge, the components of ECS may be a system that, under the appropriate conditions, produces synergy with established therapeutic agents in different diseases particularly autoimmune inflammatory diseases.

Currently, there are no clinical data indicating that the use of these ligands as adjuvant or cotreatment could improve the efficacy of the available agents or reduce the dosage thereby reducing the adverse effects and maximizing efficacy. Thus, such clinical comparisons would be very interesting and more research should be directed towards the potential synergism and antagonism of cannabinoid ligands in pharmacotherapeutics. The potential of the ECS in a wide range of disorders has been demonstrated; therefore, it is tempting and reasonable to speculate that the nature derived small molecules modulating cannabinoid receptors will have to demonstrate therapeutic efficacy and elucidate underlying potential mechanism of therapeutic benefits by cannabinoids. Additionally, lack of toxicity along with additional anxiolytic activity which appears with synthetic CB_1_ receptor antagonists, the phytocannabinoids, can potentially be promising for future armamentarium of the cannabinoid based therapeutics. The data on acute and chronic toxicity and safety is also desired in order to undergo the translation of the observed experimental benefits into humans.

The medicinal plants are part of diet since civilization and therefore based on the evidences of cannabimimetic activity of many more plants could be promoted for inclusion in the diet as these could indirectly exert immunomodulatory, nonpsychoactive, and anti-inflammatory action. This could potentially modulate inflammatory and other pathophysiological processes. The wide availability, easy accessibility, high lipophilicity, and wide therapeutic window make them an excellent candidate for therapeutic intervention. Further, the isolation and characterization of pharmacophores from these plants may provide a model for drug leads using combinatorial chemistry and* in silico* approaches for future drug discovery. These plants may also offer dietary means of treatment for targeting of endocannabinoid dysregulation or the diseases where endocannabinoid modulation represents an important therapeutic target.

The development of new drugs remains an important task for the pharmaceutical industry. The natural compounds from these herbs could provide a rich source in the search for new candidates targeting GPCRs in particular cannabinoid receptors and ECS. Developing phytocannabinoids possess cannabimimetic activity and being devoid of psychotropic activity will enhance their therapeutic spectrum. To explore this possibility, several herb-based natural compound library and cell-based cannabinoid receptor assays were developed to perform high throughput screening. We believe that the process of assay development for cannabinoid receptors, compound screening using these assays, and hit compounds identification will lead to a successful compound for future therapeutic use.

## Figures and Tables

**Table 1:** The physicochemical properties and common and IUPAC name of lead compounds modulating cannabinoid receptors.

Molecule & IUPAC name	Chemical properties	Common name(s)
Amyrin (3S,4aR,6aR,6bS,8aR,12aR,14aR,14bR)-4,4,6a,6b,8a,11,11,14b-Octamethyl-1,2,3,4a,5,6,7,8,9,10,12,12a,14,14a-tetradecahydropicen-3-ol	M. Wt.: 426.71 [g/mol] M. formula: C_30_H_50_O XLogP3-AA: 9.2 H-bond donor/acceptor: 1/1	Olean-12-en-3-beta-ol

Auroglaucin 2-[(1E,3E,5E)-Hepta-1,3,5-trienyl]-3,6-dihydroxy-5-(3-methylbut-2-enyl)benzaldehyde	M. Wt.: 298.37618 [g/mol] M. formula: C_19_H_22_O_3_ XLogP3-AA: 5.4 H-bond donor/acceptor: 2/3	Auroglaucine

Biochanin A 5,7-Dihydroxy-3-(4-methoxyphenyl)chromen-4-one	M. Wt.: 284.26 [g/mol] M. formula: C_16_H_12_O_5_ XLogP3-AA: 3 H-bond donor/acceptor: 2/5	5,7-Dihydroxy-4′-methoxyisoflavone

Betulinic acid (1R,3aS,5aR,5bR,7aR,9S,11aR,11bR,13aR,13bR)-9-Hydroxy-5a,5b,8,8,11a-pentamethyl-1-prop-1-en-2-yl-1,2,3,4,5,6,7,7a,9,10,11,11b,12,13,13a,13b-hexa-decahydrocyclopenta[a]chrysene-3a-carboxylic acid	M. Wt.: 456.70 [g/mol] M. formula: C_30_H_48_O_3_ XLogP3-AA: 8.2 H-bond donor/acceptor: 2/3	3*β*-Hydroxy-20(29)-lupaene-28-oic acid

Celastrol (2R,4aS,6aR,6aS,14aS,14bR)-10-Hydroxy-2,4a,6a,6a,9,14a-hexamethyl-11-oxo-1,3,4,5,6,13,14,14b-octahydropicene-2-carboxylic acid	M. Wt.: 450.60962 [g/mol] M. formula: C29H38O4 XLogP3: 5.9 H-bond donor/acceptor: 2/4	Celastrol; tripterine; tripterin; celastrol, *Celastrus scandens*

Beta-caryophyllene (1R,4E,9S)-4,11,11-Trimethyl-8-methylidenebicyclo[7.2.0]undec-4-ene	M. Wt.: 204.35 [g/mol] M. formula: C_15_H_24_ XLogP3-AA: 4.4 H-bond donor/acceptor: 0/0	(−)-trans-Caryophyllene

Chelerythrine 1,2-Dimethoxy-12-methyl-[1,3]benzodioxolo[5,6-c]phenanthridin-12-ium	M. Wt.: 348.37 [g/mol] M. formula: C_21_H_18_NO_4_^+^ XLogP3-AA: 4.6 H-bond donor/acceptor: 0/4	1,2-Dimethoxy-12-methyl(1,3)benzodioxolo(5,6-c)phenanthridinium

Curcumin (1E,6E)-1,7-Bis(4-hydroxy-3-methoxyphenyl)hepta-1,6-diene-3,5-dione	M. Wt.: 368.37 [g/mol] M. formula: C_21_H_20_O_6_ XLogP3-AA: 3.2 H-bond donor/acceptor: 2/6	Diferuloylmethane

Cyanidin 2-(3,4-Dihydroxyphenyl)chromenylium-3,5,7-triol	M. Wt.: 287.24 [g/mol] M. formula: C_15_H_11_O_6_^+^ H-bond donor/acceptor: 5/5	Cyanidol, 3,5,7,3′,4′-pentahydroxyflavylium

Cyanidin-3,5-di-O-glucoside (2S,3R,4S,5S,6R)-2-[2-(3,4-Dihydroxyphenyl)-7-hydroxy-3-[(2S,3R,4S,5S,6R)-3,4,5-trihydroxy-6-(hydroxymethyl)oxan-2-yl]oxychromenylium-5-yl]oxy-6-(hydroxymethyl)oxane-3,4,5-triol	M. Wt.: 611.52 [g/mol] M. formula: C_27_H_31_O_16_^+^ H-bond donor/acceptor: 11/15	Cyanin, cyanidin 3,5-O-diglucoside

Cyanidin-3-O-glucoside 2-(3,4-Dihydroxyphenyl)-5-hydroxy-3-[(3R,4S,5S,6R)-3,4,5-trihydroxy-6-(hydroxymethyl)oxan-2-yl]oxychromen-7-one	M. Wt.: 448.37 [g/mol] M. formula: C_21_H_20_O_11_ XLogP3-AA: −1.2 H-bond donor/acceptor: 7/11	—

Cyanidin 3-galactoside (2S,5R)-2-[2-(3,4-Dihydroxyphenyl)-5,7-dihydroxychro-menylium-3-yl]oxy-6-(hydroxymethyl)oxane-3,4,5-triol	M. Wt.: 449.38 [g/mol] M. formula: C_21_H_21_O_11_^+^ H-bond donor/acceptor: 8/10	Idaein, cyanidin 3-O-galactoside

Cyanidin 3-O-rutinoside 2-[[6-[2-(3,4-Dihydroxyphenyl)-5,7-dihydroxy-chromenylium-3-yl]oxy-3,4,5-trihydroxyoxan-2-yl]methoxy]-6-methyloxane-3,4,5-triol chloride	M. Wt.: 630.97 [g/mol] M. formula: C_27_H_31_ClO_15_ H-bond donor/acceptor: 10/15	Meralop, 3-O-rutino sylcyanidin,7,4′-dihydroxyflavilium chloride

Delphinidin 2-(3,4,5-Trihydroxyphenyl)chromenylium-3,5,7-triol	M. Wt.: 303.24 [g/mol] M. formula: C_15_H_11_O_7_^+^ H-bond donor/acceptor: 6/6	3,3′,4′,5,5′,7-Hexahydroxyflavylium

Euphol (3S,5R,10S,13S,14S,17S)-4,4,10,13,14-Pentamethyl-17-[(2R)-6-methylhept-5-en-2-yl]-2,3,5,6,7,11,12,15,16,17-decahydro-1H-cyclopenta[a]phenanthren-3-ol	M. Wt.: 426.71 [g/mol] M. formula: C_30_H_50_O XLogP3-AA: 8.9 H-bond donor/acceptor: 1/1	Eupha-8,24-dienol

18*β*-Glycyrrhetinic acid (2S,4aS,6aR,6aS,6bR,10S,12aS,14bR)-10-Hydroxy-2,4a,6a,6b,9,9,12a-heptamethyl-13-oxo-3,4,5,6,6a,7,8,8a,10,11,12,14b-dodecahydro-1H-picene-2-carboxylic acid	M. Wt.: 470.68 [g/mol] M. formula: C_30_H_46_O_4_ XLogP3-AA: 6.4 H-bond donor/acceptor: 2/4	18*β*-Glycyrrhetic acid, glycyrrhetinic acid

Grenadamide (5E)-N-[(E)-10-Chloro-4,6-dimethyl-5-oxodec-9-en-2-yl]-5-(chloromethylidene)octanamide	M. Wt.: 404.4141 [g/mol] M. formula: C_21_H_35_Cl_2_NO_2_ XLogP3-AA: 6.1 H-bond donor/acceptor: 1/2	—

Guineensine (2E,4E,12E)-13-(1,3-Benzodioxol-5-yl)-N-(2-methylpropyl)trideca-2,4,12-trienamide	M. Wt.: 383.52 [g/mol] M. formula: C_24_H_33_NO_3_ XLogP3-AA: 6.8 H-bond donor/acceptor: 1/3	Pipyahyine

Leucettamol A (2S,3R,5Z,8Z,11Z,14Z,17Z,20Z,28R,29S)-2,29-Diaminotriaconta-5,8,11,14,17,20-hexaene-3,28-diol	M. Wt.: 472.74 [g/mol] M. formula: C_30_H_52_N_2_O_2_ XLogP3-AA: 6.2 H-bond donor/acceptor: 4/4	—

Magnolol 2-(2-Hydroxy-5-prop-2-enylphenyl)-4-prop-2-enylphenol	M. Wt.: 266.33 [g/mol] M. formula: C_18_H_18_O_2_ XLogP3-AA: 5 H-bond donor/acceptor: 2/2	5,5′-Diallyl-2,2′-dihydroxybiphenyl

Methylhonokiol 2-(4-Methoxy-3-prop-2-enylphenyl)-4-prop-2-enylphenol	M. Wt.: 280.36 [g/mol] M. formula: C_19_H_20_O_2_ XLogP3-AA: 5.3 H-bond donor/acceptor: 1/2	4′-Methoxy-3′,5-di-2-propenyl-(1,1′-biphenyl)-2-ol,4-methoxyhonokiol

Pristimerin Methyl (2R,4aS,6aR,6aS,14aS,14bR)-10-hydroxy-2,4a,6a,6a,9,14a-hexamethyl-11-oxo-1,3,4,5,6,13,14,14b-octahydropicene-2-carboxylate	M. Wt.: 464.63 [g/mol] M. formula: C_30_H_40_O_4_ XLogP3-AA: 6.3 H-bond donor/acceptor: 1/4	24-Nor-D:A-friedooleana-1(10),3,5,7-tetraen-29-oic acid

Quercetin 2-(3,4-Dihydroxyphenyl)-3,5,7-trihydroxychromen-4-one	M. Wt.: 302.23 [g/mol] M. formula: C_15_H_10_O_7_ XLogP3: 1.5 H-bond donor/acceptor: 5/7	—

Resveratrol 5-[(E)-2-(4-Hydroxyphenyl)ethenyl]benzene-1,3-diol	M. Wt.: 228.24 [g/mol] M. formula: C_14_H_12_O_3_ XLogP3-AA: 3.1 H-bond donor/acceptor: 3/3	3,4′,5-Trihydroxystilbene

Rutamarin 2-[6-(2-Methylbut-3-en-2-yl)-7-oxo-2,3-dihydrofuro[3,2-g]chromen-2-yl]propan-2-yl acetate	M. Wt.: 356.41 [g/mol] M. formula: C_21_H_24_O_5_ XLogP3-AA: 4.4 H-bond donor/acceptor: 0/5	—

Salvinorin A Methyl (2S,4aR,6aR,7R,9S,10aS,10bR)-9-acetyloxy-2-(furan-3-yl)-6a,10b-dimethyl-4,10-dioxo-2,4a,5,6,7,8,9,10a-octahydro-1H-benzo[f]isochromene-7-carboxylate	M. Wt.: 432.46 [g/mol] M. formula: C_23_H_28_O_8_ XLogP3-AA: 2.5 H-bond donor/acceptor: 0/8	Divinorin A

Sanguinarine	M. Wt.: 332.32 [g/mol] M. formula: C_20_H_14_NO_4_^+^ XLogP3-AA: 4.4 H-bond donor/acceptor: 0/4	Dimethylene dioxybenzphenanthridine

*γ*-Sanshool (2E,4E,8Z,10E,12E)-N-Propan-2-yltetradeca-2,4,8,10,12-pentaenamide	M. Wt.: 259.38 [g/mol] M. formula: C_17_H_25_NO XLogP3-AA: 4.3 H-bond donor/acceptor: 1/1	—

Miconioside B (2S)-7-[(2S,4S,5S)-6-[[(2R,3S)-3,4-Dihydroxy-4-(hydroxylmethyl)oxolan-2-yl]oxymethyl]-3,4,5-tri-hydroxyoxan-2-yl]oxy-5-hydroxy-2-(4-hydroxy-phenyl)-6,8-dimethyl-2,3-dihydrochromen-4-one	M. Wt.: 594.56 [g/mol] M. formula: C_28_H_34_O_14_ XLogP3-AA: −0.5 H-bond donor/acceptor: 8/14	Farrerol 7-O-beta-D-apiofuranosyl(1->6)-beta-D-glucopyranoside

Piperine (2E,4E)-5-(1,3-Benzodioxol-5-yl)-1-piperidin-1-ylpenta-2,4-dien-1-one	M. Wt.: 285.33 [g/mol] M. formula: C_17_H_19_NO_3_ XLogP3: 3.5 H-bond donor/acceptor: 0/3	1-Piperoylpiperidine

Malvidin 2-(4-Hydroxy-3,5-dimethoxyphenyl)chromenylium-3,5,7-triol	M. Wt.: 331.29 [g/mol] M. formula: C_17_H_15_O_7_^+^ H-bond donor/acceptor: 4/6	3′,5′-Dimethoxy-3,4′,5,7-tetrahydroxy flavylium acid anion

Matteucinol 5,7-Dihydroxy-2-(4-methoxyphenyl)-6,8-dimethyl-2,3-dihydrochromen-4-one	M. Wt.: 314.33 [g/mol] M. formula: C_18_H_18_O_5_ XLogP3-AA: 3.4 H-bond donor/acceptor: 2/5	(2S)-5,7-Dihydroxy-2-(4-methoxyphenyl)-6,8-dimethyl-2,3-dihydro-4H-chromen-4-one

Monocillin II (4E,8E,11S)-15-Hydroxy-11-methyl-12-oxabicyclo[12.4.0]octadeca-1(14),4,8,15,17-pentaene-3,13-dione	M. Wt.: 300.349 [g/mol] M. formula: C_18_H_20_O_4_ XLogP3-AA: 4.1 H-bond donor/acceptor: 1/4	—

Nonivamide N-[(4-Hydroxy-3-methoxyphenyl)methyl]nonanamide	M. Wt.: 293.40 [g/mol] M. formula: C_17_H_27_NO_3_ XLogP3-AA: 4.2 H-bond donor/acceptor: 2/3	N-Vanillyl pelargonamide, pelargonic acid vanillylamide

Peonidin 2-(4-Hydroxy-3-methoxyphenyl)chromenylium-3,5,7-triol	M. Wt.: 301.27 [g/mol] M. formula: C_16_H_13_O_6_^+^ H-bond donor/acceptor: 4/5	3,4′,5,7-Tetrahydroxy-3′-methoxyflavylium

Pelargonidin 2-(4-Hydroxyphenyl)chromenylium-3,5,7-triol	M. Wt.: 271.24 [g/mol] M. formula: C_15_H_11_O_5_^+^ H-bond donor/acceptor: 4/4	3,4′,5,7-Tetrahydroxy flavylium chloride

Radicicol	M. Wt.: 364.77698 [g/mol] M. formula: C_18_H_17_ClO_6_ XLogP3-AA: 3.4 H-bond donor/acceptor: 2/6	Monorderne, radisico, melanotetan II, monorden A

Sciadonic acid (5E,11E,14E)-Icosa-5,11,14-trienoic acid	M. Wt: 306.48 [g/mol] M. formula: C_20_H_34_O_2_ XLogP3-AA: 6.7 H-bond donor: 1/2	Icosa-5,11,14-trienoic acid, 5c,11c,14c-eicosatrienoic acid

Semiplenamides A (2E,6E)-N-(2-Hydroxyethyl)-2-methylicosa-2,6-dienamide	M. Wt.: 365.59 [g/mol] M. formula: C_23_H_43_NO_2_ XLogP3-AA: 7.7 H-bond donor/acceptor: 2/2	—

Semiplenamides B 2-[[(2E,6E)-2-Methylicosa-2,6-dienoyl]amino]ethyl acetate	M. Wt.: 407.62 [g/mol] M. formula: C_25_H_45_NO_3_ XLogP3-AA: 8.3 H-bond donor/acceptor: 1/3	—

Semiplenamides G 2-[[(2S,3R)-2-Methyl-3-pentadecyloxirane-2-carbonyl]amino] propyl acetate	M. Wt.: 411.61 [g/mol] M. formula: C_24_H_45_NO_4_ XLogP3-AA: 7.6 H-bond donor/acceptor: 1/4	—

Serinolamide A (E)-N-[(2R)-1-Hydroxy-3-methoxypropan-2-yl]-N-methyloctadec-4-enamide	M. Wt.: 383.6083 [g/mol] M. formula: C_23_H_45_NO_3_ XLogP3-AA: 6.6 H-bond donor/acceptor: 1/3	(4E)-N-[(2R)-1-Hydroxy-3-methoxy-2-propanyl]-N-methyl-4-octadecenamide

Voacamine	M. Wt.: 704.89 [g/mol] M. formula: C_43_H_52_N_4_O_5_ XLogP3-AA: 6.1 H-bond donor/acceptor: 2/7	Voacanginine, voacamine

Yangonin 4-Methoxy-6-[(E)-2-(4-methoxyphenyl)ethenyl]pyran-2-one	M. Wt.: 258.26 [g/mol] M. formula: C_15_H_14_O_4_ XLogP3-AA: 2.7 H-bond donor/acceptor: 0/4	4-Methoxy-6-(*β*-(p-anisyl)vinyl)-*α*-pyrone

The XLogP3-AA data, molecular weight, molecular formula, and H-bond donor/H-bond were collected from NCBI, http://www.ncbi.nlm.nih.gov/pccompound/?term.

**Table 2:** The cannabinoid receptor affinity, potency, and activity of lead molecules.

Compound	CB receptor mediated effect	CB receptor affinity/potency	References
*γ*-Sanshool	Diabetes	CB_2_ agonist	Dossou et al. 2013 [106]

4′-O-Methylhonokiol	Alzheimer's diseases	CB_2_ agonist	Gertsch and Anavi-Goffer 2012 [87] Schuehly et al. 2011 [85]

Yangonin	Anxiety	CB_1_ receptor antagonist	Ligresti et al. 2012 [112]

Amyrin	Neuropathic pain	CB_1_/CB_2_ agonist, MAGL inhibitor	Simão da Silva et al. 2011 [33]

Betulinic acid	Cancer	CB_1_ antagonist/CB_2_ agonist	Liu et al. 2012 [38]

*β*-Caryophyllene	Ulcerative colitis Alzheimer's diseases Insulin resistance Alcohol addiction Anxiety Depression Nephrotoxicity Cerebral ischemia	CB_2_ agonist	Bento et al. 2011 [40] Horváth et al. 2012 [47] Al Mansouri et al. 2014 [41] Bahi et al. 2014 [49] Choi et al. 2013 [43] Klauke et al. 2014 [50] Suijun et al. 2014 [44] Guo et al. 2014 [42] Gertsch et al. 2008 [18]

Celastrol	Neuropathic pain	CB_2_ agonist	Yang et al. 2014 [55]

Chelerythrine	Neuropathic pain Neuroblastoma	CB_1_ antagonist	Lim et al. 2003 [57]

Curcumin	Neuroprotective liver fibrosis	CB_1_ antagonist/CB_2_ agonist	Hassanzadeh and Hassanzadeh, 2012 [64]

Euphol	Neuropathic pain	CB_1_/CB_2_ agonist, MAGL inhibitor	Dutra et al. 2012 [71]

18*β*-Glycyrrhetinic acid	Obesity	CB_1_ antagonist	Park et al. 2014 [73]

Pristimerin	Pain & inflammation	MAGL inhibitor	Chicca et al. 2012 [34]

Salvinorin A	Anxiety Depression Neuropathic pain Ulcerative colitis	CB_1_ agonist, FAAH inhibitor	Fichna et al. 2012 [102] Aviello et al. 2011 [100] Capasso et al. 2008 [98] Braida et al. 2009 [95] Braida et al. 2007 [99]

Malyngamide B	Inflammation	CB_1_/CB_2_ agonist	Montaser et al. 2012 [82]

Rutin	Depression	CB_1_ agonist	Su et al. 2014 [83]

Serinolamide B	Inflammation Cancer	CB_1_ and CB_2_ receptors action	Montaser et al. 2012 [82]

**Table 3:** The chemical class of compounds showing nature derived cannabinoid ligands.

Alkaloids	Terpenes and terpenoid	Polyphenols	Fatty acid derivatives
(i) Auroglaucin (ii) Chelerythrine (iii) Guineensine (iv) Bibenzyls (v) Isoperrottetin A (vi) Sanguinarine (vii) *γ*-Sanshool (viii) Voacamine (ix) 3,6-Oxidovoacangine (x) 5-Hydroxy-3,6-oxidovoacangine (xi) Haplosamates (xii) Desulfohaplosamates (xiii) Piperine (xiv) Neocosmosins (xv) Monocillins (xvi) Radicicol (xvii) Yangonin	(i) Amyrin (ii) Betulinic acid (iii) *β*-Caryophyllene (iv) Celastrol (v) Euphol (vi) Falcarinol (vii) 18*β*-Glycyrrhetinic acid (viii) Isoperrottetin A (ix) Pristimerin (x) Salvinorin A (xi) Thujone (xii) Yangonin (xiii) Thujone	(i) Biochanin A (ii) Curcumin and derivatives (iii) Cyanidin derivatives (iv) Desmodianones (v) Delphinidin (vi) (+)-Catechin derivatives (vii) Honokiol derivatives (viii) Peonidin (ix) Pelargonidin (x) Magnolol (xi) Malvidin (xii) Rutin (xiii) 6-Methyltetrapterol A (xiv) Magnolol (xv) Miconioside (xvi) Resveratrol	(i) Dodeca-2E,4E,8Z,10Z-tetraenoic acid isobutylamide (ii) Dodeca-2E,4E-dienoic acid isobutylamide (iii) 1-[(2*E*,4*E*,8*Z*)-Tetradecatrienoyl] piperidine (iv) Dodeca-2*E*,4*E*-dienoic acid isobutylamide (v) Tetradeca-2*E*,4*E*-dienoic acid isobutylamide (vi) Tetradeca-2*E*,4*E*,8*Z*-trienoic acid isobutylamide (vii) 1-[(2*E*,4*E*,8*Z*)-Tetradecatrienoyl] piperidine (viii) Malyngamides (ix) Serinolamides (x) Sciadonic acid (xi) Semiplenamides

**Table 4:** The cannabinoid receptor affinity, potency, and activity of medicinal plants.

Medicinal plants	CB mediated effect	CB affinity/potency	References
*Corydalis yanhusuo *	Neuropathic pain	CB_1_ antagonist	Huang et al. 2010 [119]
*Echinacea purpurea*	Immunomodulation	CB_2_ agonist	Chicca et al. 2009 [25]
*Linum usitatissimum*	Inflammation	CB_2_ agonist	Styrczewska et al. 2012 [120]
*Melilotus suaveolens*	Lung injury	CB_2_ agonist	Liu et al. 2014 [121]
*Morinda citrifolia*	Immunomodulation	CB_1_ antagonist/CB_2_ agonist	Palu et al. 2008 [122]
*Nelumbo nucifera*	Obesity	CB_2_ agonist	Velusami et al. 2013 [123]
*Olea europaea*	Colon cancer	CB_1_ agonist	Cotrim et al. 2012 [124]
*Rubus coreanus*	Osteoporosis	CB_1_ antagonist/CB_2_ agonist	Lim et al. 2015 [125]
*Ruta graveolens*	Diabetes	CB_2_ agonist	Rollinger et al. 2009 [126]
